# “Nothing to see here”: No structural brain differences as a function of the *Big Five* personality traits from a systematic review and meta-analysis

**DOI:** 10.1017/pen.2021.5

**Published:** 2022-08-09

**Authors:** Yen-Wen Chen, Turhan Canli

**Affiliations:** 1 Program in Integrative Neuroscience, Department of Psychology, Stony Brook University, Stony Brook, NY, USA; 2 Department of Psychiatry, Stony Brook University, Stony Brook, NY, USA

**Keywords:** Personality, Five-factor model, Brain structure, Systematic review, Meta-analysis

## Abstract

Personality reflects social, affective, and cognitive predispositions that emerge from genetic and environmental influences. Contemporary personality theories conceptualize a Big Five Model of personality based on the traits of neuroticism, extraversion, agreeableness, conscientiousness, and openness to experience. Starting around the turn of the millennium, neuroimaging studies began to investigate functional and structural brain features associated with these traits. Here, we present the first study to systematically evaluate the entire published literature of the association between the Big Five traits and three different measures of brain structure. Qualitative results were highly heterogeneous, and a quantitative meta-analysis did not produce any replicable results. The present study provides a comprehensive evaluation of the literature and its limitations, including sample heterogeneity, Big Five personality instruments, structural image data acquisition, processing, and analytic strategies, and the heterogeneous nature of personality and brain structures. We propose to rethink the biological basis of personality traits and identify ways in which the field of personality neuroscience can be strengthened in its methodological rigor and replicability.

Personality traits reflect stable emotional and motivational individual attributes and predispositions (Eysenck, 1967; McCrae & Costa, 1997). Particularly, influential models of personality derived their structure from descriptive terms that led to the formulation of the five-factor model (FFM) (Costa & McCrae, 1992; McCrae & John, 1992), which includes the traits of neuroticism, extraversion, agreeableness, conscientiousness, and openness to experience (“openness”) and which have been well-replicated across ages and cultures (Allemand et al., 2007; McCrae & Terracciano, 2005). Personality traits have been associated with various internalizing and externalizing psychopathologies. For example, neuroticism, extraversion, and conscientiousness have been robustly reported to be positively or negatively associated with depressive, anxiety, and substance use disorders among clinically diagnosed populations (Kotov et al., 2010), as well as among non-clinical populations (Hyatt et al., 2019), whereas agreeableness and conscientiousness were reported to be negatively associated with aggressive and antisocial behaviors (Vize et al., 2018).

The emergence of non-invasive imaging technologies that could be sensitive to individual differences in functional and structural brain features gave rise to neuroimaging-based personality neuroscience (Canli, 2006; Canli & Amin, 2002). For example, Canli et al. (2001, 2002) reported differential associations of neuroticism and extraversion in response to negative and positive emotional stimuli in frontal and subcortical regions, respectively, using task-based functional magnetic resonance imaging (fMRI). More recently, Yang et al. (2021) reported negative association between neuroticism and frontal activation using an implicit emotional paradigm. The Big Five personality model (Costa & McCrae, 1992; McCrae & John, 1992) received particular attention in the emergent field. Adelstein et al. (2011) reported non-overlapping functional networks as a function of different Big Five personality traits using resting-state fMRI. In one of the earliest structural imaging studies, Omura et al. (2005) examined gray matter volume (GMV) and gray matter concentration (GMC) as a function of the Big Five traits. More recently, Hyatt et al. (2019) further revealed differential associations between the Big Five and different brain structural indices, including GMV, cortical thickness (CT), and cortical surface area (SA).

As the personality neuroscience imaging literature grew, the number of mixed and highly heterogeneous findings began to accumulate. However, to our knowledge, only few studies attempted to systematically summarize the relevant literature. Montag et al. (2013) observed highly heterogeneous associations between neuroticism and regional brain structures using a qualitative review approach. Lai et al. (2019) observed highly heterogeneous associations between extraversion and regional GMV using a qualitative systematic review approach.

Specific contributors to heterogeneous study results may reflect differences in methodologies (e.g., measurement, image data acquisition and processing, statistical approach), as well as differences in the selection of study samples (Hu et al., 2011; Montag, Reuter, et al., 2013).

Age may be one sample-related variable affecting brain measures of personality traits. Although on the one hand Big Five personality traits are thought to be relatively stable over time (Terracciano et al., 2010; Wängqvist et al., 2015), studies have pointed to age-related cross-sectional differences, as well as within-individual longitudinal changes over time (Allemand et al., 2007; Donnellan & Lucas, 2008; Roberts & Mroczek, 2008), such that aging is associated with increased agreeableness and conscientiousness, and with reduced neuroticism, extraversion, and openness.

Sex may be another sample-related variable affecting brain measures of personality traits. For example, a large population-based study (Soto et al., 2011) showed that females, on average, reported higher levels of neuroticism, extraversion, agreeableness, and conscientiousness than did males in mid-adolescence and middle adulthood, whereas openness showed the opposite sex trend, with males reporting slightly higher trait levels from early adulthood.

Indeed, both age- and sex-related group differences were observed in brain structures. For example, studies reported smaller regional GMV in frontal, temporal, and parietal regions among older, relative to the younger, cohorts (Good et al., 2001; Gur et al., 2002; Smith et al., 2007), and Potvin et al. (2017, 2018) suggested that age is an important predictor for regional volumes. On the other hand, studies with regard to sex differences in brain structures are mixed. For example, a large-scale study with more than 5,000 participants reported greater regional GMV in the isthmus of the cingulate gyrus in males and greater regional GMV in the superior parietal lobule in females (Ritchie et al., 2018); however, inconsistent observations from other studies have been noted (Eliot et al., 2021). In summary, both age and sex have been suggested to be associated with Big Five personality traits and brain structures; therefore, they could potentially contribute to the association between personality and brain structure.

Considering the large and heterogeneous neuroimaging literature on the Big Five, and only a small number of attempts to apply analytic tools to identify sources of heterogeneity in this literature, we set out to conduct a comprehensive systematic and meta-analytic review of the structural imaging literature on the Big Five, to identify and quantify sources of variance, and to derive from this analysis our prescription for strengthening the field of personality neuroscience going forward.

## 1. Method

### 1.1. Literature search strategy

The current systematic review and meta-analysis were conducted following the Preferred Reporting Items for Systematic Reviews and Meta-Analyses guidelines (PRISMA) (Liberati et al., 2009). The present review searched PubMed, PsycInfo, and Web of Science up to March 4, 2020, using the following search terms: (*personality OR “personality trait” OR “personality traits” OR “big five” OR “big five personality” OR neuroticism OR extraversion OR agreeableness OR openness OR conscientiousness OR “five-factor personality model” OR “NEO-Five Factor Inventory” OR “NEO-FFI” OR “five factor model”*) AND (*MRI OR “magnetic resonance imaging” OR “structural magnetic resonance imaging” OR “structural MRI” OR “gray matter volume” OR GMV OR VBM OR “voxel-based morphometry” OR SBM OR “surface-based morphometry” OR “cortical thickness” OR “cortical thinning” OR “cortical surface area” OR “surface area” OR “cortical folding” OR “brain structure” OR neuroimaging OR “brain imaging” OR imaging*). To limit the search results to human-related studies, we used the term “*human*” as a filter, if applicable. In addition, we manually searched articles from relevant reviews and reference lists. Two coders (Y.-W. C. and R. A.) independently conducted the literature search, the selection of eligible studies, and study result recording for meta-analysis studies. The results of included studies and data recording were compared, and a consensus was reached through discussion for any inconsistencies.

### 1.2. Literature inclusion and exclusion criteria

To be included in this systematic review, the inclusion criteria were (1) peer-reviewed empirical studies, (2) published in English, (3) measuring self-reported Big Five personality traits, (5) measuring gray matter volume (GMV), cortical thickness (CT), and/or surface area (SA) using T1-weighted structural MRI, (6) measuring and reporting the association between Big Five traits and brain structural measures (including non-significant results), (7) results from cross-sectional data (if a study had more than one time point, only results from one time point (baseline results were prioritized if applicable) would be included). In addition to the abovementioned criteria, to be included in the meta-analysis, a study needed to (1) conduct whole-brain voxel-based morphometry (VBM) or surface-based morphometry (SBM), (2) the same threshold was applied throughout the whole brain within each study, (3) report result coordinates of significant clusters in Montreal Neurological Institute (MNI) or Talairach space. Based on the sample sizes of previous structural neuroimaging meta-analyses using SDM (Du et al., 2014; Kimmel et al., 2016; Lai et al., 2019; Li et al., 2020; Wise et al., 2017) and the recommended minimum of 10 studies to adequately test the heterogeneity of meta-analysis results (Sterne et al., 2011), we only conducted meta-analyses on samples of at least 10 studies, as was the case for GMV studies, but not CT (*n* = 9 across five traits) and SA (*n* = 6 across five traits) studies. In addition to the Big Five global traits, we also included results from trait facets, based on the NEO-Personality Inventory-Revised (NEO-PI-R) (Costa & McCrae, 1992).

### 1.3. Data recording for meta-analysis

We first reviewed and recorded demographic characteristics (e.g., sample size, number of females and males, age of the sample (mean, standard deviation, and range), whether participants came from a larger project/data pool), and behavioral and imaging methodological information, such as personality instruments, image acquisition parameters (e.g., scanner magnetic field strength, voxel size), image processing methodologies (e.g., VBM or SBM, smoothing kernel), and statistical analyses (e.g., statistical threshold, correction of whole-brain multiple comparison). Second, we recorded the results for meta-analysis, including coordinates of significant clusters and measures of effect size (e.g., *t* scores, *Z* scores, and/or *p* values) (Albajes-Eizagirre, Solanes, Fullana, et al., 2019).

### 1.4. Quality assessment

The present study used a 10-item checklist to assess the quality of each study included in the meta-analysis. The checklist was adopted and modified from previous structural imaging studies (Brambilla et al., 2003; Du et al., 2014; Li et al., 2020). Assessed items included the reported characteristics of the participants, behavioral and imaging methodologies, results, and discussion (detailed description for items was listed in Table S1). Each item was scored 1, 0.5, or 0 if the study fulfilled, partially fulfilled, or did not fulfil the criteria, respectively, and only studies with summed scores greater than 6 were included in the meta-analysis (Li et al., 2020). Note that this checklist is not designed as an assessment tool nor as a standardized measurement; however, it provides an objective indication of the rigor and transparency of each study. The inter-rater reliability of quality assessment for meta-analysis studies was assessed with intraclass correlation coefficient (ICC), and good reliability between two coders was observed (*ICC* = .91).

### 1.5. Meta-analysis

We conducted the meta-analysis using seed-based *d* mapping with permutation of subject images software (SDM-PSI, version 6.21) (Albajes-Eizagirre, Solanes, & Radua, 2019; Albajes-Eizagirre, Solanes, Fullana, et al., 2019; Albajes-Eizagirre, Solanes, Vieta, et al., 2019) (the software is publicly available at https://www.sdmproject.com/). The SDM-PSI procedure included the followings steps: First, it used the coordinates and effect size (*t* values) of the cluster peaks that showed association with personality traits. If a study provided *Z*, *r*, or *p* values instead of *t* values, we first converted them into corresponding *t* values. Note that if a study did not report the exact effect size, according to SDM-PSI manual recommendation, the study’s threshold (*p* value) was then used as conservative measure of the effect size. Second, during preprocessing step, SDM-PSI converts the effect sizes into Hedge’s g and the peak coordinates and effect sizes were used to create maps of the lower and upper bounds of the potential effect sizes for each study. We used the existing mask from SDM-PSI to restrict the analysis to gray matter. Third, SDM-PSI estimated the likely effect size and its standard error using maximum likelihood estimation and multiple imputations for each study. For the group analysis, SDM-PSI conducted a random-effects model, which was weighted by the sample size, within- and between-study heterogeneity. We set the imputation at the default of 20. Finally, SDM-PSI used Rubin’s rules to combine the imputed datasets. We used the commonly used SDM threshold (voxel-wise *p* < .005, *SDM*-Z > 1, cluster size greater than 10 voxels) and presented the result peak clusters in MNI coordinates.

#### 1.5.1. Heterogeneity testing for potential moderators

To explore the potential moderators in the relation between Big Five traits and GMV, we conducted a series of heterogeneity tests. First, to investigate the heterogeneities in sample population, GMV processing, and Big Five personality instruments, we conducted a series of sub-group analyses by excluding (1) patient studies; (2) studies measuring gray matter density; (3) studies measuring T1w/T2w ratio; (4) studies preprocessing without segmentation; (5) studies using non-NEO instruments. We used the same threshold as in the main meta-analysis (voxel-wise *p* < .005, *SDM*-Z > 1, cluster size greater than 10 voxels) for these sub-group analyses. Second, to investigate the heterogeneities in sample characteristics, structural image data acquisition and processing strategies, statistical approach and statistical significance threshold, and quality, we conducted a series of meta-regression analyses with the following variables: Mean age of the samples, percentage of females, scanner magnetic field strength, processing method (VBM (as reference) *versus* SBM), smooth kernel, threshold correction (uncorrected *versus* cluster-level correction *versus* voxel-/vertex-level correction), covariates used in the analysis, and quality scores. Meta-regression in SDM-PSI examines whether a moderator correlates with the values of the activation voxels (i.e., robust peaks across studies). It uses permutation at the study level and implements the Freedman-Lane procedure for optimal parameter estimates (Albajes-Eizagirre et al., 2019; Freedman & Lane, 1983; Winkler et al., 2014). We used the commonly used threshold for meta-regression (voxel-wise *p* < .0005, *SDM*-Z > 1, cluster size greater than 10 voxels) to minimize the probability of false positives (Lai, Wang, Zhao, Zhang, et al., 2019; Li et al., 2020).

## 2. Result

### 2.1. Included studies and study characteristics: General description

**Figure 1.**
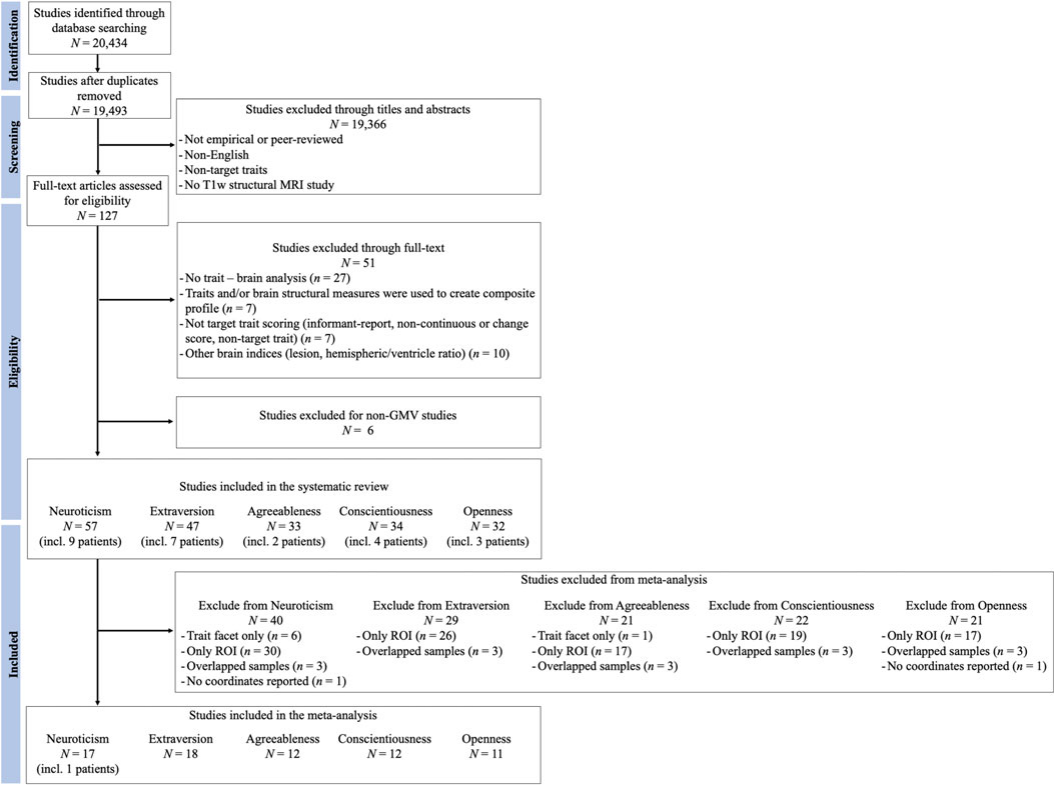
Flowchart of Gray Matter Volume Literature Search and Selection of Eligible Studies. The Flowchart was modified from Liberati et al. (2009).

**Figure 2.**
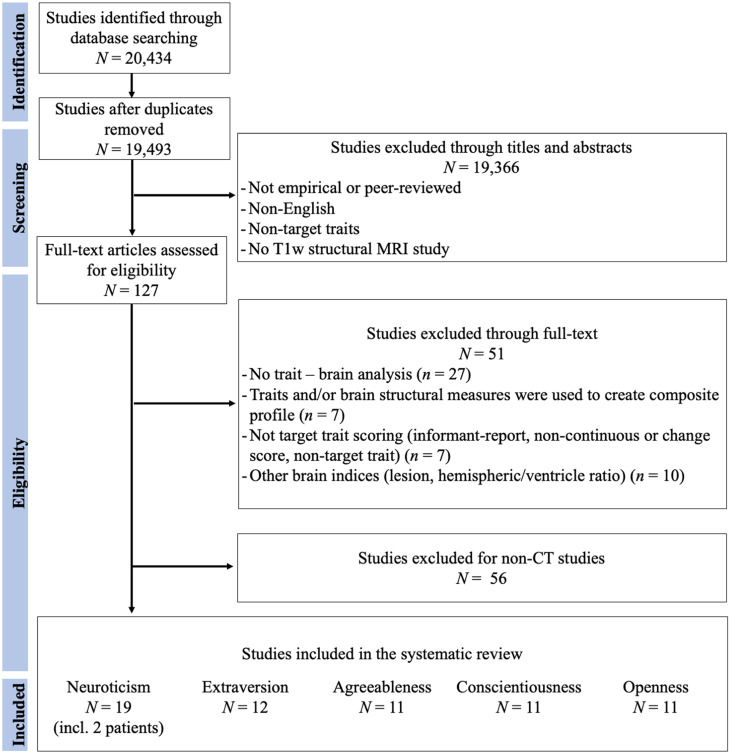
Flowchart of Cortical Thickness Literature Search and Selection of Eligible Studies. The Flowchart was modified from Liberati et al. (2009).

**Figure 3.**
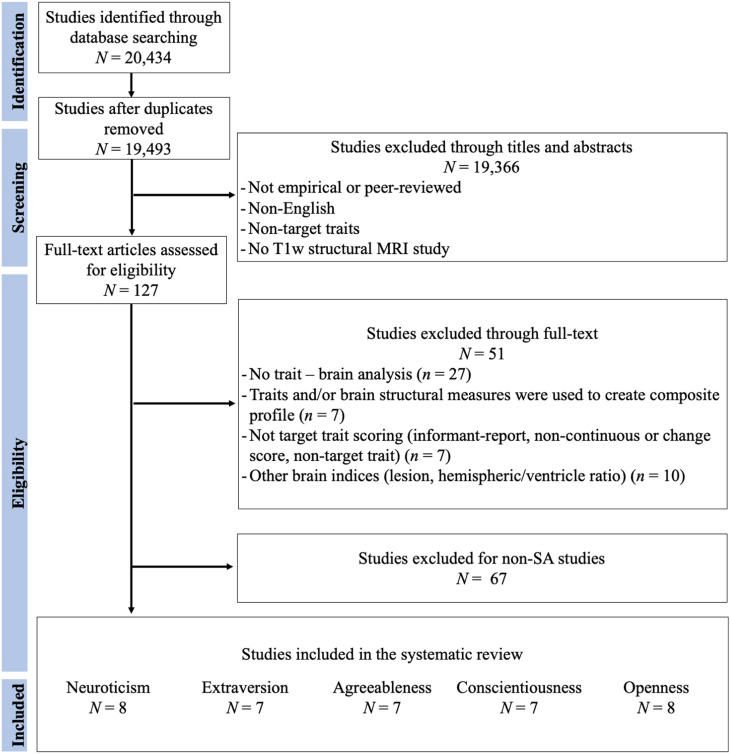
Flowchart of Surface Area Literature Search and Selection of Eligible Studies. The Flowchart was modified from Liberati et al. (2009).

Figures 1–3 represent the flowcharts of the process of the literature search and selection of eligible studies. Across three databases (PubMed, PsycInfo, and Web of Science), the search resulted in 19 493 unique articles, of which 127 articles were eligible for the full article assessment across five personality traits and three brain indices. After full article assessment, across five traits, we identified 70 studies of GMV, 20 studies of CT, and 9 studies of SA. The sample characteristics of the studies included in the systematic review and meta-analysis are summarized in Table 1. The full list of included studies can be found in the supplementary materials.

**Table 1.** Summary of studies included in the systematic review and meta-analysis

Trait	# Studies	Sample (median, range)	Female/Male	Mean Age (range)
Meta-Analysis Gray Matter Volume				
Neuroticism	17	3340 (87, 37–1104)	1878/1462	33.75 (17–85)
Extraversion	18	3099 (68, 30–1104)	1726/1373	33.4 (17–85)
Agreeableness	12	2760 (97.5, 37–1104)	1562/1198	36.32 (17–85)
Conscientiousness	12	3148 (132, 37–1104)	1750/1398	35.43 (17–85)
Openness	11	2784 (116, 37–1104)	1583/1201	37.74 (17–85)
Systematic Review Gray Matter Volume				
Neuroticism	51	15 563 (102.5, 19–1870)	8613/6826	33.81 (12–90)
Extraversion	47	11 928 (87, 19–1870)	6520/5284	34.43 (12–88)
Agreeableness	32	10 769 (112, 19–1870)	5781/4615	32.6 (12–85)
Conscientiousness	34	11 136 (113, 19–1870)	6140/4872	35.08 (12–88)
Openness	32	10 686 (125.5, 19–1870)	5886/4676	33.14 (12–85)
Systematic Review Cortical Thickness				
Neuroticism	19	7944 (223, 14–1106)	4485/3459	36.25 (14–85)
Extraversion	12	4048 (165.5, 14–1104)	2359/1689	37.74 (14–85)
Agreeableness	11	5016 (265, 28–1104)	2889/2127	36.93 (14–85)
Conscientiousness	11	5016 (265, 28–1104)	2889/2127	36.93 (14–85)
Openness	11	4184 (223, 28–1104)	2427/1757	36.31 (14–85)
Systematic Review Surface Area				
Neuroticism	8	4825 (542.5, 56–1106)	2676/2149	38.27 (17–85)
Extraversion	7	3719 (507, 56–1104)	2076/1643	38.27 (17–85)
Agreeableness	7	3719 (507, 56–1104)	2076/1643	38.27 (17–85)
Conscientiousness	7	3719 (507, 56–1104)	2076/1643	38.27 (17–85)
Openness	8	3904 (386, 56–1104)	2170/1734	36.24 (16–85)

*Note.* Among the systematic review studies, some studies (Joffe et al., 2009; Nair et al., 2016; Yang, Hou, et al., 2017) did not report the demographic information for the final sample; this was the case when, for example, only some participants contributed personality trait and/or structural image data, but a study only reported the demographic information for the total sample.

### 2.2. Study result reporting principles

To provide a comprehensive summary of the literature, we listed all results reported from each study, including results from alternative analytic approaches within the same study. However, to avoid double-counting the results from the same study using multiple alternative analytic approaches, the summaries presented in this Result section follow the principles listed here: (1) If a study had both whole-brain and ROI approaches, results from the whole-brain analysis were prioritized; (2) If a study used both voxel- or vertex-based ROI and mean ROI approaches, results from voxel- or vertex-based analysis were prioritized; (3) If a study reported results from both corrected and uncorrected thresholds, results from corrected threshold were prioritized; (4) If a study included more than one group, results from the largest sample size within that study were prioritized.

The following sections below summarize systematic review and meta-analysis (only applicable for GMV studies) results separately by each of the Big Five personality traits for GMV, CT, and SA. If none of the included studies measured the specific associations, such as trait facets, global brain indices, it would be denoted as “None” in the corresponding result section.

### 2.3. Neuroticism

#### 2.3.1. Gray matter volume studies

Study characteristics of the included studies are displayed in Table 2 (meta-analysis studies) and Table S2 (systematic review studies). In addition, Table S17 summarizes patient studies across five traits and three brain indices.

**Table 2.** Characteristics of gray matter volume meta-analysis studies with neuroticism (17 studies)

Study	Sample Size (F/M)	Mean Age (Range)	Personality Instrument	Image Process (Software)	Covariate	Threshold (Correction)	Results (*t* range)
Cremers et al., 2011	65 (42/23)	40.5 (21–56)	NEO-FFI	VBM (SPM5)	Age, TGMV, alternative trait (E), scan centers	0.05 (voxel, FEW/Uncor)	*Cor*: NSig *Uncor:* (*t* 3.77–4.11)
							*Pos.* l MTG
							*Neg.* r SMA
DeYoung et al., 2010	116 (58/58)	22.9 (18–40)	NEO-PI-R	VBM (BIS)	Age, sex, TBV, other four traits	0.05 (cluster, MCS)	**(study without segmentation)** (*t* NS)
							*Pos.* l/r MCG, l MTG, r CRB
							*Neg.* l BG, r medFG, r PRC
Du et al., 2014	298 (158/140)	19.9 (17–27)	NEO-PI-R	VBM (SPM8)	Age, gender, TBV	0.05 (cluster, NSTA)	(*t* 5.03)
							*Pos.* dACC
							*Neg.* NSig
Hu et al., 2011	62 (31/31)	26.6 (20–40)	NEO-FFI	VBM (SPM5)	Age, gender, TGMV	0.05 (voxel, FWE)	NSig
Kapogiannis et al., 2013	87 (42/45)	72.0 (59–85)	NEO-PI-R	VBM (SPM8)	* This study performed a conjunction analysis between two time points	0.05 (cluster, FWE)	(*t* 3.51–5.23)
					Age (both time points), sex (both time points), ICV (Time 1), education (both time points), same trait (another time point), other four traits (both time points), time 1 and time 2		*Pos.* r LNG, r FSF, r MOG, r PRC, r CAL
							*Neg.* r infOFC, r RLO, r MFG, r PHC, r MTG
Koelsch et al., 2013	59 (34/25)	19.9 (18–27)	NEO-FFI	VBM (SPM8)	Age, gender, TBV	0.05 (voxel, FWE)	NSig
Lewis et al., 2018	578 (268/310)	72.7 (NS)	50-IPIP	SBM (CIVET 1.1.12)	Age, sex, ICV, other four FFM traits, intelligence	0.05 (vertex, FDR)	NSig
T. Li et al., 2017	108 (64/44)	40.3 (19–60)	NEO-FFI	SBM (CCS)	Age, sex, ICV	0.05 (voxel, FDR)	NSig
Liu et al., 2013	227 (168/59)	25.8 (18–62)	NEO-FFI	VBM (SPM8)	Age, sex, other four traits, scanner type	0.05 (cluster, FWE)	NSig
Lu et al., 2014	71 (37/34)	22.4 (19–26)	EPQ-RSC	VBM (SPM8)	Age, sex, ICV	0.05 (cluster, MCS)	(*t* 3.36–3.46)
							*Pos.* r CRB
							*Neg.* l SFG
Omura et al., 2005	41 (22/19)	23.8 (NS)	NEO-PI-R	VBM (SPM2)	Age, sex	0.001 (Uncor, k > 200)	**(GMC)** (*t* 4.19–4.73)
							*Pos.* NSig
							*Neg.* r SPL, r ANG
Owens et al., 2019	1104 (599/505)	28.8 (22–37)	NEO-FFI	SBM (FS 5.3)	Age, sex, ICV, other four traits	0.05 (cluster, MCS)	(*t* 2.93–4.22)
							*Pos.* NSig
							*Neg.* l STG, l/r CNS, l LOG, r DLPFC
Privado et al., 2017	56 (56/0)	18.3 (17–22)	NEO-FFI	SBM (CIVET 2.0)	TGMV	0.01 (vertex, FWE)	*Cor:* NSig *Uncor (p < .05):* (*t* 2.27–2.45)
							*Pos.* NSig
							*Neg.* l PRC, l SOG
Rivera-Bonet et al., 2019	57 TLE (36/21)	34.4 (18–50)	NEO-FFI	SBM (HCP-PP)	Age, gender, ICV, laterality of seizure focus	0.01 (cluster, MCS)	(*t* 2.64–3.98)
							*Pos*. NSig
							*Neg*. l SFG, l AINS, l PRC, l LPOC
Taki et al., 2013	274 (161/113)	51.2 (21–80)	NEO-PI-R	VBM (SPM8)	Age, gender, ICV, other four traits	0.05 (voxel, FWE)	NSig
Yasuno et al., 2017	37 (10/27)	28.1 (21–45)	NEO-FFI	VBM (SPM12)		0.05 (cluster, FWE)	**(T1w/T2w)** NSig
Zou et al., 2018	100 (50/50)	21.9 (18–27)	EPQ-RSC	VBM (SPM8)	Age, sex, head motion	0.05 (cluster, FWE)	NSig

*Note.* Threshold: The threshold denotes the threshold p value, correction level (voxel-/vertex- *versus* cluster-level), and correction method used in the study; Result *t* range: The range of the absolute *t* value for significant peaks reported by each study was presented in the Result column. If only one value was presented, it suggests only one significant peak from the study result. For studies with effect sizes as “NS (not specified),” meta-analysis was conducted with converting the threshold used in the study. Abbreviation used in the table can be found in Supplementary Table S20.

##### 2.3.1.1. Gray matter volume systematic review: Global gray matter indices

Of the 51 included studies, nine studies examined the association between neuroticism and global GMV indices, including total GMV (TGMV), total brain volume (TBV), neocortical volume (NCV), and total brain/gray matter ratio (TBR/TGMR), five studies reported negative associations with TGMV (Jackson et al., 2011; Liu et al., 2013; Tuerk et al., 2016) and TBV (Bjørnebekk et al., 2013; Knutson et al., 2001), and four studies reported no association with TGMV (Benedict et al., 2013; Cremers et al., 2011; Knutson et al., 2001; Kunz et al., 2017) and NCV (Benedict et al., 2008). Moreover, two additional studies that measured the specific facets of neuroticism (depression, anxiety) reported no association with TGMV and TBV (Schutter et al., 2017; Wei et al., 2015).

##### 2.3.1.2. Gray matter volume systematic review: Regional gray matter volume

Of the 51 included studies, 11 whole-brain analysis (WBA) studies reported no association between neuroticism and regional GMV, including one study that used a T1w/T2w ratio signal (Yasuno et al., 2017), and 19 ROI analysis studies reported no association between neuroticism and regional GMV, including one study that used gray matter density (GMD) (Lai, Wang, Zhao, Qiu, et al., 2019), one study that examined GMV as a function of BDNF genotype (Joffe et al., 2009), and five patient cohort studies (Hayano et al., 2009; Lee et al., 2016; Moayedi et al., 2011; Nair et al., 2016; Nickson et al., 2016). Of the 19 studies that reported significant associations, negative associations were consistently reported (i.e., two or more studies reporting significant associations for the same region) for the bilateral OFC, SFG and right MFG, left STG, left LOG, and left CNS (note that the last two regions were from studies with non-independent samples: Hyatt et al., 2019; Owens et al., 2019; Riccelli et al., 2017), and positive associations were consistently reported for the left AMY. Associations in opposite directions were reported across studies for the right PRC, left MTG, right FSF, and right CRB.

##### 2.3.1.3. Gray matter volume systematic review: Neuroticism facets

Of the 51 included studies, three studies that examined these six facets of neuroticism. Six additional studies examined one or more specific facets, without examining the global neuroticism trait. Study characteristics and results of neuroticism facets are displayed in Table S2. Of the five studies examining the anxiety facet, two studies reported negative associations with TBV (Bjørnebekk et al., 2013; Knutson et al., 2001), and three studies reported no association with global or regional GMV (Schutter et al., 2012; Weber et al., 2010; Wei et al., 2015). Of the seven studies examining the depression facet, five studies reported negative associations with TBV (Bjørnebekk et al., 2013) and regional GMV in frontal, ACC, THA, and CRB (Gustin et al., 2013; Schutter et al., 2012) and positive associations in frontal, HIP, and INS (Gustin et al., 2013; Yang et al., 2016; Yang, Yin, et al., 2017) (note that two studies from Yang et al. might include non-independent samples from the same project data), and two studies reported no association with global or regional GMV (Knutson et al., 2001; Weber et al., 2010). Of the three studies examining the Self-consciousness facet, two studies reported negative associations with TBV (Bjørnebekk et al., 2013; Knutson et al., 2001), and one study reported no association with regions GMV (Weber et al., 2010). Of the three studies examining the impulsiveness facet, only one study reported a negative association with TBV. Of the three studies examining the vulnerability facet, one study reported a negative association with TBV (Bjørnebekk et al., 2013) and another study reported a positive association with left AMY (Weber et al., 2010). Of the three studies examining the hostility facet, only one study reported a negative association with TBV (Bjørnebekk et al., 2013).

##### 2.3.1.4. Gray matter volume meta-analysis

To examine robustness and replication of the results across studies, we conducted a meta-analysis using SDM-PSI, independently for each of the Big Five traits. Neuroticism and GMV studies are summarized in Table 2. Seventeen studies were included in the meta-analysis of neuroticism and GMV, including one patient cohort study (Rivera-Bonet et al., 2019), one study that measured GMD (Lai, Wang, Zhao, Qiu, et al., 2019), one study that used a T1w/T2w ratio signal (Yasuno et al., 2017), and one study that did not use segmentation (DeYoung et al., 2010). Four studies (Nostro et al., 2017; Owens et al., 2019; Riccelli et al., 2017; Toschi & Passamonti, 2019) that met the meta-analysis criteria included non-independent samples, as all four studies used data from Human Connectome Project (HCP), and therefore, only the study with the largest sample size (i.e., Owens et al., 2019) was included in the meta-analysis. One study (Tuerk et al., 2016) that met the meta-analysis criteria was not included because we were unable to obtain region coordinates from the publication or its authors; thus, this study was excluded from the meta-analysis, leaving 17 studies for meta-analytic study. The meta-analysis revealed no robustly significant regions associated with neuroticism (mean Hedge’s *g* = 0.0048 (−0.1077 – 0.0883)), although we observed a negative trend (at *p* < .05, *k* > 10, *SDM-Z* > 1) in the right SFG.

#### 2.3.2. Cortical thickness studies

Study characteristics of the included studies are displayed in Table S7. In addition, Table S17 summarizes patient studies across five traits and three brain indices.

##### 2.3.2.1. Cortical thickness systematic review: Global cortical thickness indices

Of the 19 included studies, only one study (Sweeney et al., 2019) examined the association between neuroticism and global CT measure and reported no association between neuroticism and total CT.

##### 2.3.2.2. Cortical thickness systematic review: Regional cortical thickness

Of the 19 included studies, four WBA studies reported no association between neuroticism and regional CT and four ROI analysis studies reported no association between neuroticism and regional CT. Of the 11 studies that reported significant associations, positive associations were consistently reported for the left SFG and MFG (note that both two regions were from studies with non-independent samples: Castagna, 2019; Hyatt et al., 2019; Riccelli et al., 2017; Zhu et al., 2020). No negative associations were consistently reported for the same region across studies. On the other hand, associations in opposite directions were reported across studies for the right SFG. In addition, two studies comparing patients and healthy participants reported opposite associations for the left INS and right FSF (Zhao et al., 2017), and left OFC (Moayedi et al., 2011).

##### 2.3.2.3. Cortical thickness systematic review: Neuroticism facets

None.

#### 2.3.3. Surface area studies

Study characteristics of the included studies are displayed in Table S12.

##### 2.3.3.1. Surface area systematic review: Global surface area indices

None.

##### 2.3.3.2. Surface area systematic review: Regional surface area

Of the eight included studies, three WBA studies reported no association between neuroticism and regional SA, but no ROI analysis studies reported association between neuroticism and regional SA. Of the 5 studies that reported significant associations, negative associations were consistently reported for the bilateral SFG, left LOG, and left CNS (note that all those regions were from studies with non-independent samples: Castagna, 2019; Hyatt et al., 2019; Owens et al., 2019; Riccelli et al., 2017; Zhu et al., 2020). No positive associations were consistently reported for the same region across studies. No association in opposite directions was reported across studies.

##### 2.3.3.3. Surface area systematic review: Neuroticism facets

Of the eight included studies, one study conducted a post hoc facet analysis to determine the contribution of six facets and reported that the anxiety, depression, and vulnerability to stress facets had the greatest contribution to the negative association between global neuroticism and cingulate, frontal, and temporal GMV (Bjørnebekk et al., 2013) (Table S12). We identified no additional study that examined neuroticism facets without examining global neuroticism.

### 2.4. Extraversion

#### 2.4.1. Gray matter volume studies

Study characteristics of the included studies are displayed in Table 3 (meta-analysis studies) and Table S3 (systematic review studies). In addition, Table S17 summarizes patient studies across five traits and three brain indices.

**Table 3.** Characteristics of gray matter volume meta-analysis studies with extraversion (18 studies)

Study	Sample size (F/M)	Mean Age (Range)	Personality Instrument	Image Process (Software)	Covariate	Threshold (Correction)	Results (*t* range)
Andari et al., 2014	30 (13/17)	23.6 (18–37)	NEO-PI-R	VBM (SPM2)	Age, sex, TGMV	0.05 (cluster, NSTA)	(*t* 3.36–6.17)
							*Pos.* NSig
							*Neg.* l MTG, r ITG, r PCN, r LNG, r PHC, r HIP
Coutinho et al., 2013	52 (29/23)	25.0 (19–52)	NEO-FFI	VBM (SPM8)	Age, gender, ICV	0.05 (cluster, MCS)	(*t* 4.02–4.41)
							*Pos.* NSig
							*Neg.* l/r MFG, l/r midOFC, r IFG, r SFG
Cremers et al., 2011	65 (42/23)	40.5 (21–56)	NEO-FFI	VBM (SPM5)	Age, TGMV, alternative trait (N), scan lefts	0.05 (voxel, FWE) 0.001 (Uncor, k > 25)	(*t* 3.49–4.50) *Cor*: NSig *Uncor:*
							*Pos.* l/r medOFC, l PCG, l CRB, r AMY, r SPL
							*Neg.*
DeYoung et al., 2010	116 (58/58)	22.9 (18–40)	NEO-PI-R	VBM (BIS)	Age, sex, TBV, other four traits	0.05 (cluster, MCS)	**(study without segmentation)** (*t* NS)
							*Pos.* r medOFC
							*Neg.* Nsig
Forsman et al., 2012	32 (0/32)	33.2 (19–49)	16PF (version 5)	VBM (SPM2)	Age	0.05 (voxel, FDR)	(*t* 3.66–5.82)
							*Pos.* NSig
							*Neg.* l/r SFG, l VLPFC, l/r SMG, l IPS, l IOG, l THA, r MFG, r SFS, r IFG, r ANG, r MTG/ITG, r CAD
Hu et al., 2011	62 (31/31)	26.6 (20–40)	NEO-FFI	VBM (SPM5)	Age, gender, TGMV	0.05 (voxel, FWE)	NSig
Kapogiannis et al., 2013	87 (42/45)	72.0 (59–85)	NEO-PI-R	VBM (SPM8)	* This study performed a conjunction analysis between two time points	0.05 (cluster, FWE)	(*t* 3.80–5.60)
					Age (both time points), sex (both time points), ICV (Time 1), education (both time points), same trait (another time point), other four traits (both time points), time 1 and time 2		*Pos.* l STG, l ACG, l/r MFG, l SMA, l IOG, l SFG, r INS
							*Neg.* l IOG, l SPL, r PHC
Koelsch et al., 2013	59 (34/25)	19.9 (18–27)	NEO-FFI	VBM (SPM8)	age, gender, TBV	0.05 (voxel, FWE)	NSig
Lewis et al., 2018	578 (268/310)	72.7 (NS)	50-IPIP	SBM (CIVET 1.1.12)	Age, sex, ICV, other four traits, intelligence	0.05 (vertex, FDR)	NSig
T. Li et al., 2017	108 (64/44)	40.3 (19–60)	NEO-FFI	SBM (CCS)	Age, sex, ICV	0.05 (voxel, FDR)	NSig
Liu et al., 2013	227 (168/59)	25.8 (18–62)	NEO-FFI	VBM (SPM8)	Age, sex, other four traits, scanner type	0.05 (cluster, FWE)	NSig
Lu et al., 2014	71 (37/34)	22.4 (19–26)	EPQ-RSC	VBM (SPM8)	Age, sex, ICV	0.05 (cluster, MCS)	(*t* 3.22–5.11)
							*Pos.* NSig
							*Neg.* l/r AMY, l/r PHC, l SFG, r MTG
Omura et al., 2005	41 (22/19)	23.8 (NS)	NEO-PI-R	VBM (SPM2)	Age, sex	0.001 (Uncor, k > 200)	**(GMC)** (*t* 4.17–5.17)
							*Pos*. l/r OFC, l FSF, l PHC, r CAL, r STG
							*Neg*. l/r PRC
Owens et al., 2019	1104 (599/505)	28.8 (22–37)	NEO-FFI	SBM (FS 5.3)	Age, sex, ICV, other four traits	0.05 (cluster, MCS)	(*t* 3.38)
							*Pos.* r PRC
							*Neg.* NSig
Privado et al., 2017	56 (56/0)	18.3 (17–22)	NEO-FFI	SBM (CIVET 2.0)	TGMV	0.01 (vertex, FWE)	(*t* 3.92)
							*Pos.* l MOG
							*Neg.* NSig
Taki et al., 2013	274 (161/113)	51.2 (21–80)	NEO-PI-R	VBM (SPM8)	Age, gender, ICV, other four traits	0.05 (voxel, FWE)	NSig
Yasuno et al., 2017	37 (10/27)	28.1 (21–45)	NEO-FFI	VBM (SPM12)		0.05 (cluster, FWE)	**(T1w/T2w)** NSig
Zou et al., 2018	100 (50/50)	21.9 (18–27)	EPQ-RSC	VBM (SPM8)	Age, sex, head motion	0.05 (cluster, FWE)	(*t* 4.05–4.06)
							*Pos.* NSig
							*Neg.* l/r PUT

Note. Threshold: The threshold denotes the threshold p value, correction level (voxel-/vertex- versus cluster-level), and correction method used in the study; Result t range: The range of the absolute t value for significant peaks reported by each study was presented in the Result column. If only one value was presented, it suggests only one significant peak from the study result. For studies with effect sizes as “NS (not specified),” meta-analysis was conducted with converting the threshold used in the study. Abbreviation used in the table can be found in Supplementary Table S20.

##### 2.4.1.1. Gray matter volume systematic review: Global gray matter indices

Of the 47 included studies, nine studies examined the association between extraversion and global GMV indices. Two studies reported a positive association with TGMV (Cremers et al., 2011; Kunz et al., 2017), one study reported a positive association with NCV among patients with MS (Benedict et al., 2008), and six studies reported no association with TGMV (Benedict et al., 2013; Forsman et al., 2012; Jackson et al., 2011; Knutson et al., 2001; Liu et al., 2013) or TBV (Bjørnebekk et al., 2013; Knutson et al., 2001).

##### 2.4.1.2. Gray matter volume systematic review: Regional gray matter volume

Of the 47 included studies, nine WBA studies reported no association between extraversion and regional GMV, including one study that used a T1w/T2w ratio signal (Yasuno et al., 2017), and 16 ROI analysis studies reported no association between extraversion and regional GMV, including one study that examined GMV as a function of BDNF genotype (Joffe et al., 2009) and five patient cohort studies (Lee et al., 2016; Nair et al., 2016; Nickson et al., 2016; Onitsuka et al., 2005; Weber et al., 2010). Of the 20 studies that reported significant associations, negative associations were consistently reported for the left DLPFC, right IFG, right MTG, and right PHC. No positive associations were consistently reported for the same region across studies. On the other hand, associations in opposite directions were reported across studies for a wide set of regions, including frontal (bilateral OFC and MFG, left SFG, right PRC), temporal (left STG and PHC), left SPL, left IOG, and subcortical (bilateral PUT and right CAD) regions.

##### 2.4.1.3. Gray matter volume systematic review: Extraversion facets

Of the 47 included studies, two studies examined these six facets of extraversion. We identified no additional study that examined extraversion facets without measuring the global extraversion trait. Study characteristics and results of extraversion facets are displayed in Table S3. Of the two studies that examined facets of extraversion, one study reported no association between the six extraversion facets and any of the prior defined regional GMV among patients with EOD (Weber et al., 2010), and another study reported that the gregariousness facet had the greatest contribution to the positive association between global extraversion and bilateral CAD (M. Li et al., 2019).

##### 2.4.1.4. Gray matter volume meta-analysis

Extraversion and GMV studies are summarized in Table 3. Eighteen studies were included in the meta-analysis of extraversion and GMV, including one study that measured GMD (Lai, Wang, Zhao, Qiu, et al., 2019), one study that used a T1w/T2w ratio signal (Yasuno et al., 2017), and one study that did not use segmentation (DeYoung et al., 2010). Four studies (Nostro et al., 2017; Owens et al., 2019; Riccelli et al., 2017; Toschi & Passamonti, 2019) that met meta-analysis criteria included non-independent samples, as all four studies used data from HCP, and therefore, only the study with the largest sample size (Owens et al., 2019) was included in the meta-analysis. Meta-analysis with all included 18 studies revealed no robustly significant regions associated with extraversion (mean Hedge’s *g* = 0.0038 (−0.0445 – 0.0855)), even at a more liberal threshold (*p* < .05, *k* > 10, *SDM-Z* > 1).

#### 2.4.2. Cortical thickness studies

Study characteristics of the included studies are displayed in Table S8. In addition, Table S17 summarizes patient studies across five traits and three brain indices.

##### 2.4.2.1. Cortical thickness systematic review: Global cortical thickness indices

None.

##### 2.4.2.2. Cortical thickness systematic review: Regional cortical thickness

Of the 12 included studies, three WBA studies reported no association between extraversion and regional CT and three ROI analysis studies reported no association between extraversion and regional CT. Of the six studies that reported significant associations, no positive or negative associations were consistently reported for the same region across studies, nor associations in opposite directions were reported across studies.

##### 2.4.2.3. Cortical thickness systematic review: Extraversion facets

Of the 12 included studies, only one study conducted a post hoc facet analysis and reported that the excitement-seeking facet had the greatest contribution to the negative association between global extraversion and IFG CT (Bjørnebekk et al., 2013) (Table S8). We identified no additional study that examined extraversion facets without examining global extraversion was identified.

#### 2.4.3. Surface area studies

Study characteristics of the included studies are displayed in Table S13.

##### 2.4.3.1. Surface area systematic review: Global surface area indices

None.

##### 2.4.3.2. Surface area systematic review: Regional surface area

Of the seven included studies, four WBA studies reported no association between extraversion and regional SA, but no ROI analysis studies reported association between extraversion and regional SA. Of the three studies that reported significant associations, no positive or negative associations were consistently reported for the same region across studies, nor associations in opposite directions were reported across studies.

##### 2.4.3.3. Surface area systematic review: Extraversion facets

None.

### 2.5. Agreeableness

#### 2.5.1. Gray matter volume studies

Study characteristics of the included studies are displayed in Table 4 (meta-analysis studies) and Table S4 (systematic review studies). In addition, Table S17 summarizes patient studies across five traits and three brain indices.

**Table 4.** Characteristics of gray matter volume meta-analysis studies with agreeableness (12 studies)

Study	Sample size (F/M)	Mean Age (Range)	Personality Instrument	Image Process (Software)	Covariate	Threshold (Correction)	Results (*t* range)
Coutinho et al., 2013	52 (29/23)	25.0 (19–52)	NEO-FFI	VBM (SPM8)	Age, gender, ICV	0.05 (cluster, MCS)	(*t* 3.94–4.10)
							*Pos.* NSig
							*Neg.* l MOG, l PCG, r IPG
DeYoung et al., 2010	116 (58/58)	22.9 (18–40)	NEO-PI-R	VBM (BIS)	Age, sex, TBV, other four traits	0.05 (cluster, MCS)	**(study without segmentation)** (*t* NS)
							*Pos.* l PCG, r FSF
							*Neg.* r STS
Hu et al., 2011 ^MA^	62 (31/31)	26.6 (20–40)	NEO-FFI	VBM (SPM5)	Age, gender, TGMV	0.05 (voxel, FWE)	NSig
							* When only control for Age: (*t* 5.99–6.22)
							*Pos.* NSig
							*Neg.* l/r CRB, r supOFC
Kapogiannis et al., 2013	87 (42/45)	72.0 (59–85)	NEO-PI-R	VBM (SPM8)	* This study performed a conjunction analysis between two time points	0.05 (cluster, FWE)	(*t* 3.75–5.75)
					Age (both time points), sex (both time points), ICV (Time 1), education (both time points), same trait (another time point), other four traits (both time points), time 1 and time 2		*Pos.* l SPG, r inf. OFC, r midOFC, r MTP
							*Neg.* l/r medSFG, l PHC, l MFG, l STG, r CAL
Koelsch et al., 2013	59 (34/25)	19.94 (18–27)	NEO-FFI	VBM (SPM8)	Age, gender, TBV	0.05 (voxel, FWE)	NSig
Lewis et al., 2018	578 (268/310)	72.73 (NS)	50-IPIP	SBM (CIVET 1.1.12)	Age, sex, ICV, other four traits, intelligence	0.05 (vertex, FDR)	NSig
T. Li et al., 2017	108 (64/44)	40.28 (19–60)	NEO-FFI	SBM (CCS)	Age, sex, ICV	0.05 (voxel, FDR)	NSig
Liu et al., 2013	227 (168/59)	25.8 (18–62)	NEO-FFI	VBM (SPM8)	Age, sex, other four traits, scanner type	0.05 (cluster, FWE)	NSig
Owens et al., 2019	1104 (599/505)	28.8 (22–37)	NEO-FFI	SBM (FS 5.3)	Age, sex, ICV, other four traits	0.05 (cluster, MCS)	(*t* 3.40)
							*Pos.* r DLPFC
							*Neg.* NSig
Privado et al., 2017	56 (56/0)	18.29 (17–22)	NEO-FFI	SBM (CIVET 2.0)	TGMV	0.01 (vertex, FWE)	NSig
Taki et al., 2013 ^MA^	274 (161/113)	51.2 (21–80)	NEO-PI-R	VBM (SPM8)	Age, gender, ICV, other four traits	0.05 (voxel, FWE)	NSig
Yasuno et al., 2017	37 (10/27)	28.1 (21–45)	NEO-FFI	VBM (SPM12)		0.05 (cluster, FWE)	**(T1w/T2w)** NSig

*Note.* Threshold: The threshold denotes the threshold p value, correction level (voxel-/vertex- *versus* cluster-level), and correction method used in the study; Result *t* range: The range of the absolute *t* value for significant peaks reported by each study was presented in the Result column. If only one value was presented, it suggests only one significant peak from the study result. For studies with effect sizes as “NS (not specified),” meta-analysis was conducted with converting the threshold used in the study. Abbreviation used in the table can be found in Supplementary Table S20.

##### 2.5.1.1. Gray matter volume systematic review: Global gray matter indices

Of the 32 included studies and one Trust facet study, six studies examined the association between agreeableness and global GMV indices. One study reported a positive association with NCV among patients with MS (Benedict et al., 2008), four studies (Bjørnebekk et al., 2013; Knutson et al., 2001; Kunz et al., 2017; Liu et al., 2013), and one Trust facet study (Haas et al., 2015) reported no association with TGMV or TBV.

##### 2.5.1.2. Gray matter volume systematic review: Regional gray matter volume

Of the 32 included studies, nine WBA studies reported no association between agreeableness and regional GMV, including one study that used a T1w/T2w ratio signal (Yasuno et al., 2017), and 10 ROI analysis studies reported no association between agreeableness and regional GMV, including one study that used GMD (Lai, Wang, Zhao, Qiu, et al., 2019). Of the 10 studies that reported significant associations, negative associations were consistently reported for the right SFG, left MFG, left STG, left PHC, left SFG, and left MFG (note that the last two regions were from studies with non-independent samples: Hyatt et al., 2019; Riccelli et al., 2017; Zhu et al. 2020). No positive associations were consistently reported for the same region across studies. On the other hand, associations in opposite directions were reported across studies for the left posterior cingulate region.

##### 2.5.1.3. Gray matter volume systematic review: Agreeableness facets

None of the included studies examined specific facets of agreeableness. One additional study examined the Trust facet and reported positive and negative associations with regional GMV in a wide set of regions (Haas et al., 2015) (Table S4).

##### 2.5.1.4. Gray matter volume meta-analysis

Agreeableness and GMV studies are summarized in Table 4. Twelve studies were included in the meta-analysis of agreeableness and GMV, including one study that used a T1w/T2w ratio signal (Yasuno et al., 2017) and one study that did not use segmentation (DeYoung et al., 2010). Four studies (Nostro et al., 2017; Owens et al., 2019; Riccelli et al., 2017; Toschi & Passamonti, 2019) that met the meta-analysis criteria included non-independent samples, as all four studies used data from HCP, and therefore, only the study with the largest sample size (Owens et al., 2019) was included in the meta-analysis. Meta-analysis with all included 12 studies revealed no robustly significant regions associated with agreeableness (mean Hedge’s *g* = 0.0052 (−0.1212 – 0.1281)), even at a more liberal threshold (*p* < .05, *k* > 10, *SDM-Z* > 1).

#### 2.5.2. Cortical thickness studies

Study characteristics of the included studies are displayed in Table S9. In addition, Table S17 summarizes patient studies across five traits and three brain indices.

##### 2.5.2.1. Cortical thickness systematic review: Global cortical thickness indices

None.

##### 2.5.2.2. Cortical thickness systematic review: Regional cortical thickness

Of the 11 included studies, six WBA studies reported no association between agreeableness and regional CT, and one ROI analysis studies reported no association between agreeableness and regional CT. Of the four studies that reported significant associations, negative associations were consistently reported for the left SFG and MFG (note that both two regions were from studies with non-independent samples: Hyatt et al., 2019; Riccelli et al., 2017; Zhu et al. 2020). No positive associations were consistently reported for the same region across studies. No associations in opposite directions were reported across studies.

##### 2.5.2.3. Cortical thickness systematic review: Agreeableness facets

None.

#### 2.5.3. Surface area studies

Study characteristics of the included studies are displayed in Table S14.

##### 2.5.3.1. Surface area systematic review: Global surface area indices

None.

##### 2.5.3.2. Surface area systematic review: Regional surface area

Of the seven included studies, four WBA studies reported no association between agreeableness and regional SA, and one ROI analysis studies reported no association between agreeableness and regional SA. Of the two studies that reported significant associations, no positive or negative associations were consistently reported for the same region across studies, nor associations in opposite directions were reported across studies.

##### 2.5.3.3. Surface area systematic review: Agreeableness facets

None.

### 2.6. Conscientiousness

#### 2.6.1. Gray matter volume studies

Study characteristics of the included studies are displayed in Table 5 (meta-analysis studies) and Table S5 (systematic review studies). In addition, Table S17 summarizes patient studies across five traits and three brain indices.

**Table 5.** Characteristics of gray matter volume meta-analysis Studies with conscientiousness (12 studies)

Study	Sample size (F/M)	Mean Age (Range)	Personality Instrument	Image Process (Software)	Covariate	Threshold (Correction)	Results (*t* range)
Chen et al., 2018	351 (191/160)	20.0 (17 – 27)	NEO-PI-R	VBM (SPM8)	Age, gender, TGMV	0.05 (cluster, FWE)	(*t* 4.85)
							*Pos.* r IFG
							*Neg.* Nsig
DeYoung et al., 2010	116 (58/58)	22.9 (18 – 40)	NEO-PI-R	VBM (BIS)	Age, sex, TBV, other four traits	0.05 (cluster, MCS)	**(study without segmentation)** (*t* NS)
							*Pos.* l MFG
							*Neg.* r FSF
Hu et al., 2011	62 (31/31)	26.6 (20 – 40)	NEO-FFI	VBM (SPM5)	Age, gender, TGMV	0.05 (voxel, FWE)	NSig
Kapogiannis et al., 2013	87 (42/45)	72.0 (59 – 85)	NEO-PI-R	VBM (SPM8)	* This study performed a conjunction analysis between two time points	0.05 (cluster, FWE)	(*t* 3.75–6.42)
					Age (both time points), sex (both time points), ICV (Time 1), education (both time points), same trait (another time point), other four traits (both time points), time 1 and time 2		*Pos.* r/l SFG, l PCN, l FSF, l CAD, l ACG, l LNG, l HIP, r PSC, r PRC, r STG
							*Neg.* l/r medSFG, l STG, l SPL, l CAL, r MTP, r PSC, r inf. OFC
Lewis et al., 2018	578 (268/310)	72.7 (NS)	50-IPIP	SBM (CIVET 1.1.12)	Age, sex, ICV, other four FFM traits, intelligence	0.05 (vertex, FDR)	NSig
T. Li et al., 2017	108 (64/44)	40.3 (19 – 60)	NEO-FFI	SBM (CCS)	Age, sex, ICV	0.05 (voxel, FDR)	NSig
Liu et al., 2013	227 (168/59)	25.8 (18 – 62)	NEO-FFI	VBM (SPM8)	Age, sex, other four traits, scanner type	0.05 (cluster, FWE)	NSig
Owens et al., 2019	1104 (599/505)	28.8 (22 – 37)	NEO-FFI	SBM (FS 5.3)	Age, sex, ICV, other four traits	0.05 (cluster, MCS)	NSig
Privado et al., 2017	56 (56/0)	18.3 (17 – 22)	NEO-FFI	SBM (CIVET 2.0)	TGMV	0.01(vertex, FWE)	NSig
Taki et al., 2013	274 (161/113)	51.2 (21 – 80)	NEO-PI-R	VBM (SPM8)	Age, gender, ICV, other four traits	0.05 (voxel, FWE)	NSig
Wang et al., 2019	148 (60/88)	18.5 (17 – 20)	NEO-FFI	VBM (SPM12)	Age, sex, TGMV	0.05 (cluster, NSTA)	(*t* 4.84–5.18)
							*Pos.* l/r SPL
							*Neg.* r MFG
Yasuno et al., 2017	37 (10/27)	28.1 (21 – 45)	NEO-FFI	VBM (SPM12)		0.05 (cluster, FWE)	**(T1w/T2w)** NSig

*Note.* Threshold: The threshold denotes the threshold p value, correction level (voxel-/vertex- *versus* cluster-level), and correction method used in the study; Result *t* range: The range of the absolute *t* value for significant peaks reported by each study was presented in the Result column. If only one value was presented, it suggests only one significant peak from the study result. For studies with effect sizes as “NS (not specified),” meta-analysis was conducted with converting the threshold used in the study. Abbreviation used in the table can be found in Supplementary Table S20.

##### 2.6.1.1. Gray matter volume systematic review: Global gray matter indices

Of the 34 included studies, seven studies examined the association between conscientiousness and global GMV indices. All seven studies reported no association with TGMV (Benedict et al., 2013; Jackson et al., 2011; Knutson et al., 2001; Kunz et al., 2017; Liu et al., 2013), TBV (Bjørnebekk et al., 2013; Knutson et al., 2001), or NCV (Benedict et al., 2008).

##### 2.6.1.2. Gray matter volume systematic review: Regional gray matter volume

Of the 34 included studies, nine WBA studies reported no association between conscientiousness and regional GMV, including one study that used a T1w/T2w ratio signal (Yasuno et al., 2017), and 12 ROI analysis studies reported no association between conscientiousness and regional GMV, including one study that used GMD (Lai, Wang, Zhao, Qiu, et al., 2019), one study that examined GMV as a function of BDNF genotype (Joffe et al., 2009), and another that examined GMV as a function of APOE genotype (Kunz et al., 2017). Of the 11 studies that reported significant associations, no positive or negative associations were consistently reported for the same region across studies. On the other hand, associations in opposite directions were reported across studies for the left ACC and left SPL.

##### 2.6.1.3. Gray matter volume systematic review: Conscientiousness facets

None.

##### 2.6.1.4. Gray matter volume meta-analysis

Conscientiousness and GMV studies are summarized in Table 5. Twelve studies were included in the meta-analysis of conscientiousness and GMV, including one study that used a T1w/T2w ratio signal (Yasuno et al., 2017) and one study that did not use segmentation (DeYoung et al., 2010). Four studies (Nostro et al., 2017; Owens et al., 2019; Riccelli et al., 2017; Toschi & Passamonti, 2019) that met the meta-analysis criteria included non-independent samples, as all four studies used data from HCP, and therefore, only the study with the largest sample size (Owens et al., 2019) was included in the meta-analysis. Meta-analysis with all included 12 studies revealed no robustly significant regions associated with conscientiousness (mean Hedge’s *g* = 0.0040 (−0.1240 – 0.1159)), even at a more liberal threshold (*p* < .05, *k* > 10, *SDM-Z* > 1).

#### 2.6.2. Cortical thickness studies

Study characteristics of the included studies are displayed in Table S10. In addition, Table S17 summarizes patient studies across five traits and three brain indices.

##### 2.6.2.1. Cortical thickness systematic review: Global cortical thickness indices

None.

##### 2.6.2.2. Cortical thickness systematic review: Regional cortical thickness

Of the 11 included studies, five WBA studies reported no association between conscientiousness and regional CT, and two ROI analysis studies reported no association between conscientiousness and regional CT. Of the four studies that reported significant associations, positive associations were consistently reported for the right OFC. No negative associations were consistently reported for the same region across studies. No associations in opposite directions were reported across studies.

##### 2.6.2.3. Cortical thickness systematic review: Conscientiousness facets

None.

#### *2.6.3*. Surface Area Studies

Study characteristics of the included studies are displayed in Table S15.

##### 2.6.3.1. Surface area systematic review: Global surface area indices

None.

##### 2.6.3.2. Surface area systematic review: Regional surface area

Of the seven included studies, three WBA studies reported no association between conscientiousness and regional SA, but no ROI analysis studies reported association between conscientiousness and regional SA. Of the four studies that reported significant associations, negative associations were consistently reported for the left MTG (note that this region was from studies with non-independent samples: Hyatt et al., 2019; Owens et al., 2019). No positive associations were consistently reported for the same region across studies. No associations in opposite directions were reported across studies.

##### 2.6.3.3. Surface area systematic review: Conscientiousness facets

Of the seven included studies, one study conducted a post hoc facet analysis and reported that the achievement striving and self-discipline facets had the greatest contribution to the negative association between global conscientiousness and STG SA (Bjørnebekk et al., 2013) (Table S15). We identified no additional study that examined conscientiousness facets without examining global conscientiousness was identified.

### 2.7. Openness

#### 2.7.1. Gray matter volume studies

Study characteristics of the included studies are displayed in Table 6 (meta-analysis studies) and Table S6 (systematic review studies). In addition, Table S17 summarizes patient studies across five traits and three brain indices.

**Table 6.** Characteristics of gray matter volume meta-analysis studies with openness (11 studies)

Study	Sample size (F/M)	Mean Age (Range)	Personality Instrument	Image Process (Software)	Covariate	Threshold (Correction)	Results (*t* range)
DeYoung et al., 2010	116 (58/58)	22.9 (18 – 40)	NEO-PI-R	VBM (BIS)	Age, sex, TBV, other four traits	0.05 (cluster, MCS)	**(study without segmentation)** NSig
Hu et al., 2011	62 (31/31)	26.6 (20 – 40)	NEO-FFI	VBM (SPM5)	Age, gender, TGMV	0.05 (voxel, FWE)	NSig
							* When only control for TGMV: (*t* 5.31)
							*Pos.* NSig
							*Neg.* l MCG
Jauk et al., 2015	135 (84/51)	28.4 (18.08 – 55.67)	BFSI	VBM (SPM8)	Age, sex, scanner, creativity (originality, fluency), intelligence	0.05 (cluster, MCS)	(*t* 4.70)
							*Pos.* NSig
							*Neg.* r PCN
Kapogiannis et al., 2013	87 (42/45)	72.0 (59 – 85)	NEO-PI-R	VBM (SPM8)	* This study performed a conjunction analysis between two time points	0.05 (cluster, FWE)	(*t* 3.59–6.34)
					Age (both time points), sex (both time points), ICV (Time 1), education (both time points), same trait (another time point), other four traits (both time points), time 1 and time 2		*Pos.* l THA, r SFG
							*Neg.* l MFG, l STG, l PSC, l/r FSF, l IPG, l SMA, r sup. OFC, r SFG, r RCG, r PCN
Lewis et al., 2018	578 (268/310)	72.7 (NS)	50-IPIP	SBM (CIVET 1.1.12)	Age, sex, ICV, other four traits, intelligence	0.05 (vertex, FDR)	NSig
T. Li et al., 2017	108 (64/44)	40.3 (19 – 60)	NEO-FFI	SBM (CCS)	Age, sex, ICV	0.05 (voxel, FDR)	NSig
Liu et al., 2013	227 (168/59)	25.8 (18 – 62)	NEO-FFI	VBM (SPM8)	Age, sex, other four traits, scanner type	0.05 (cluster, FWE)	NSig
Owens et al., 2019	1104 (599/505)	28.8 (22 – 37)	NEO-FFI	SBM (FS 5.3)	Age, sex, ICV, other four traits	0.05 (cluster, MCS)	(*t* 2.74–4.01)
							*Pos.* l ITG, r INS
							*Neg.* NSig
Privado et al., 2017	56 (56/0)	18.3 (17 – 22)	NEO-FFI	SBM (CIVET 2.0)	TGMV	0.01 (vertex, FWE)	*Cor*: NSig *Uncor (p < .05):* (*t* 2.08–2.71)
							*Pos.* l MOG, l INS, r STG, r SPM, r FSF
							*Neg.* NSig
Taki et al., 2013	274 (161/113)	51.2 (21 – 80)	NEO-PI-R	VBM (SPM8)	Age, gender, ICV, other four traits	0.05 (voxel, FWE)	NSig
Yasuno et al., 2017	37 (10/27)	28.1 (21 – 45)	NEO-FFI	VBM (SPM12)		0.05 (cluster, FWE)	**(T1w/T2w)** (*t* 3.62–4.47)
							Pos. Nsig
							Neg. r medFG, r ACC, r INS, r PCC

Note. Threshold: The threshold denotes the threshold p value, correction level (voxel-/vertex- versus cluster-level), and correction method used in the study; Result t range: The range of the absolute t value for significant peaks reported by each study was presented in the Result column. If only one value was presented, it suggests only one significant peak from the study result. For studies with effect sizes as “NS (not specified),” meta-analysis was conducted with converting the threshold used in the study. Abbreviation used in the table can be found in Supplementary Table S20.

##### 2.7.1.1. Gray matter volume systematic review: Global gray matter indices

Of the 32 included studies, six studies examined the association between openness and global GMV indices. All six studies reported no association with TGMV (Jauk et al., 2015; Knutson et al., 2001; Kunz et al., 2017; Liu et al., 2013), TBV (Bjørnebekk et al., 2013; Knutson et al., 2001), or NCV (Benedict et al., 2008).

##### 2.7.1.2. Gray matter volume systematic review: Regional gray matter volume

Of the 32 included studies, eight WBA studies reported no association between regional GMV and openness, including one study that that did not use segmentation (DeYoung et al., 2010), and 10 ROI analysis studies reported no association between regional GMV and openness, including one study that used GMD (Lai, Wang, Zhao, Qiu, et al., 2019), one study that examined GMV as a function of BDNF genotype (Joffe et al., 2009), and one patient cohort study (Leutgeb et al., 2016). Of the 13 studies that reported significant associations, positive associations were consistently reported for the PHC, bilateral CAD, and left ITG (note that the last region was from studies with non-independent samples: Owens et al., 2019; Riccelli et al., 2017) regions, and negative associations were consistently reported for the left MFG and right ACC. On the other hand, associations in opposite directions were reported across studies for the right INS, right PCC, left STG, left PSC, and right PCN.

##### 2.7.1.3. Gray matter volume systematic review: Openness facets

None.

##### 2.7.1.4. Gray matter volume meta-analysis

Openness and GMV studies are summarized in Table 6. Eleven studies were included in the meta-analysis of openness and GMV, including one study that used a T1w/T2w ratio signal (Yasuno et al., 2017) and one study that did not use segmentation (DeYoung et al., 2010). Four studies (Nostro et al., 2017; Owens et al., 2019; Riccelli et al., 2017; Toschi & Passamonti, 2019) that met the meta-analysis criteria included non-independent samples, as all four studies used data from HCP, and therefore, only the study with the largest sample size (Owens et al., 2019) was included in the meta-analysis. One study (Vartanian et al., 2018) that met the meta-analysis criteria was not included because we were unable to obtain region coordinates from the publication or its authors; thus, this study was excluded from the meta-analysis, leaving 11 studies for meta-analytic study. Meta-analysis with all included 11 studies revealed no significant regions associated with openness (mean Hedge’s *g* = 0.0039 (−0.0914 – 0.0426)), although we observed a negative trend (*p* < .05, *k* > 10, *SDM-Z* > 1) in the right PCN was observed.

#### 2.7.2. Cortical thickness studies

Study characteristics of the included studies are displayed in Table S11. In addition, Table S17 summarizes patient studies across five traits and three brain indices.

##### 2.7.2.1. Cortical thickness systematic review: Global cortical thickness indices

None.

##### 2.7.2.2. Cortical thickness systematic review: Regional cortical thickness

Of the 11 included studies, five WBA studies reported no association between openness and regional CT, but no ROI analysis studies reported association between openness and regional CT. Of the six studies that reported significant associations, negative associations were consistently reported for the bilateral MFG, right SFG, and left SPL (note that this region was from studies with non-independent samples: Hyatt et al., 2019; Owens et al., 2019; Riccelli et al., 2017). No positive associations were consistently reported for the same region across studies. No associations in opposite directions were reported across studies.

##### 2.7.2.3. Cortical thickness systematic review: Openness facets

None.

#### 2.7.3. Surface area studies

Study characteristics of the included studies are displayed in Table S16.

##### 2.7.3.1. Surface area systematic review: Global surface area indices

None.

##### 2.7.3.2. Surface area systematic review: Regional surface area

Of the eight included studies, five WBA studies reported no association between openness and regional SA, but no ROI analysis studies reported association between openness and regional SA. Of the three studies that reported significant associations, positive associations were consistently reported for the left ITG (note that this region was from studies with non-independent samples: Hyatt et al., 2019; Owens et al., 2019; Riccelli et al., 2017). No negative associations were consistently reported for the same region across studies. No associations in opposite directions were reported across studies.

##### 2.7.3.3. Surface area systematic review: Openness facets

None.

### 2.8. Age differences

This section summarizes studies examined age differences in three brain structural indices, five personality traits, and association between personality trait and brain structural indices.

#### 2.8.1. Age differences in brain structural indices

Seven studies examined the association between age and brain structure. Table S18 summarizes studies examining age differences in GMV, CT, and/or SA.

#### 2.8.2. Age-dependent associations between the big five and brain structure

Table S18 also summarizes studies examining age differences in five personality traits and in the association between personality and brain structural indices.

##### 2.8.2.1. Neuroticism

Of the three studies that examined age-dependent associations between neuroticism and brain structure, one study reported no neuroticism x age interaction on regional GMV (Jackson et al., 2011), one study reported no age difference in association between composite neuroticism anxiety and depression facets and CRB GMV (Schutter et al., 2017), and another study reported a three-way neuroticism x age x sex interaction for the ACC CT, in which that older, relative to younger, females who reported higher levels of neuroticism had thinner CT, whereas older, relative to younger, males who reported higher levels of neuroticism had thicker CT (Sweeney et al., 2019).

##### 2.8.2.2. Extraversion

Only one study examined age-dependent associations between extraversion and brain structure and reported no extraversion x age interaction on regional GMV (Jackson et al., 2011).

##### 2.8.2.3. Agreeableness

We identified no study examined age-dependent associations between agreeableness and brain structure.

##### 2.8.2.4. Conscientiousness

Only one study examined age-dependent associations between conscientiousness and brain structure and reported a conscientiousness x age interaction for the AMY and PHC GMV, in which individuals who reported higher levels of conscientiousness had smaller age-related cross-sectional decline in GMV (Jackson et al., 2011).

##### 2.8.2.5. Openness

Only one study examined age-dependent associations between openness and brain structure and reported that GMV mediated the negative association between openness and age (Kitamura et al., 2016).

### 2.9. Sex differences

This section summarizes studies examined sex differences in three brain structural indices, five personality traits, and association between personality trait and brain structural indices.

#### 2.9.1. Sex differences in brain structural indices

Four studies examined sex differences in brain structure. Table S19 summarizes studies examining sex differences in GNV, CT, and/or SA.

#### 2.9.2. Sex-dependent associations between the big five and brain structure

Table S19 also summarizes studies examining sex differences in five personality traits and in the association between personality and brain structural indices.

##### 2.9.2.1. Neuroticism

Of the 13 studies examined sex-dependent associations between neuroticism and brain structure, three studies reported sex-dependent associations between neuroticism and regional GMV for the ACC (Blankstein et al., 2009), HIP (Montag, Eichner, et al., 2013), POS, and FSF (Nostro et al., 2017), one study reported a three-way neuroticism x age x sex interaction for the ACC CT (Sweeney et al., 2019), and eleven studies reported no sex-dependent associations between neuroticism and GMV, CT, and/or SA.

##### 2.9.2.2. Extraversion

Of the nine studies examined sex-dependent associations between extraversion and brain structure, three studies reported sex-dependent associations between extraversion and regional GMV for the ACC (Cremers et al., 2011), POS, FSF, THA, and CRB (Nostro et al., 2017), and medFG (Blankstein et al., 2009), and six studies reported no sex-dependent associations between extraversion and GMV, CT, and/or SA.

##### 2.9.2.3. Agreeableness

Of the five studies examined sex-dependent associations between agreeableness and brain structure, one study reported sex-dependent associations between agreeableness and GMV for the right CRB (Hu et al., 2011), and four studies reported no sex-dependent associations between agreeableness and GMV, CT, and/or SA.

##### 2.9.2.4. Conscientiousness

Of the six studies examined sex-dependent associations between conscientiousness and brain structure, one study reported sex-dependent associations between conscientiousness and regional GMV for the left PCN/POS (Nostro et al., 2017), and five studies reported no sex-dependent associations between conscientiousness and GMV, CT, and/or SA.

##### 2.9.2.5. Openness

Of the five studies examined sex-dependent associations between openness and brain structure, one study reported sex-dependent associations between openness and regional GMV for the supOFC (Hu et al., 2011), and four studies reported no sex-dependent associations between openness and GMV, CT, and/or SA.

### 2.10. Meta-analysis heterogeneity testing

To explore potential heterogeneities across studies, we first conducted a series of sub-group analyses excluding studies (1) with patients (*n* = 1 across five traits); (2) measuring gray matter density (*n* = 1); (3) measuring T1w/T2w ratio (*n* = 1); (4) preprocessing without segmentation (*n* = 1); (5) using non-NEO instruments (*n* = 5). With regard to the heterogeneity in Big Five instruments, the majority of studies included in the meta-analysis used the NEO (NEO-PI-R (*n* = 7) or NEO-FFI (*n* = 11)). None of the sub-group meta-analyses revealed significant results at the set threshold (*p <* .005, *k* > 10). Table 7 summarizes the mean and range of the voxel-wise effect sizes (Hedge’s *g*) for main and sub-group meta-analysis results. Second, we conducted a series of meta-regression analyses with the following variables: Mean age of the samples, the percentage of females, scanner magnetic field strength, processing method (VBM *versus* SBM), smooth kernel, threshold correction (uncorrected *versus* cluster-level correction *versus* voxel-/vertex-level correction), covariates used in the analysis, and quality scores. With regard to the heterogeneity in the covariates used in the study analysis, we examined the influence of (1) the inclusion of ICV (*n* = 8 across five traits), that is, studies that did not include ICV as covariate as the reference; (2) the inclusion of total GMV (*n* = 6), that is, studies that did not include total GMV as covariate as the reference; (3) the inclusion of any global brain indices (*n* = 17, including ICV, total GMV, and TBV), that is, studies that did not include any global brain indices as covariate as the reference; (4) the inclusion of other personality traits as covariates (*n* = 7), that is, studies that did not include other personality traits as covariate as the reference; and (5) the total number of covariates (an average of 4.4 covariates across five traits ranging from 0 to 18 covariates), respectively. All the above variables, except the total number of covariates, showed no effect on the meta-analysis results for the associations of Big Five personality traits and GMV. The total number of covariates yielded a positive significant result (*p* < .0005, *k* > 10) in conscientiousness at right superior frontal gyrus ([8, 42, 56], *SDM-Z* = 3.35, *p* = .0004, *k* = 11). However, this significant result was driven by a single study (Kapogiannis et al., 2013) that included 18 covariates. The effect of total number of covariates became non-significant after excluding this outlier study. Table 8 summarizes the mean and range of the voxel-wise effect sizes (Hedge’s *g*) for meta-regression results.

**Table 7.** Voxel-wise effect sizes (hedge’s g) of the main meta-analysis and sub-group meta-analysis results

Meta-analysis	All Included	Excluding Patient	Excluding Patient, GMD	Excluding Patient, GMD, T1w/T2w, no segmentation	Excluding Non-NEO
Neuroticism					
# Studies	17	16	15	13	14
Mean Hedge’s g (Range)	0.0048 (−0.11 – 0.09)	0.0048 (−0.12 – 0.10)	0.0049 (−0.12 – 0.10)	0.0030 (−0.04 – 0.04)	0.0058 (−0.14 – 0.12)
Extraversion					
# Studies	18	NA	NA	15	14
Mean Hedge’s g (Range)	0.0038 (−0.04 – 0.09)			0.0034 (−0.06 – 0.04)	0.0043 (−0.04 – 0.11)
Agreeableness					
# Studies	12	NA	NA	10	11
Mean Hedge’s g (Range)	0.0052 (−0.12 – 0.13)			0.0033 (−0.05 – 0.05)	0.0069 (−0.14 – 0.14)
Conscientiousness					
# Studies	12	NA	NA	10	11
Mean Hedge’s g (Range)	0.0040 (−0.12 – 0.12)			0.0031 (−0.05 – 0.05)	0.0045 (−0.14 – 0.14)
Openness					
# Studies	11	NA	NA	9	9
Mean Hedge’s g (Range)	0.0039 (−0.09 – 0.04)			0.0035 (−0.07 – 0.05)	0.0040 (−0.09 – 0.06)

*Note.* Results marked as NA indicate that the included studies did not have such characteristic. For example, only Neuroticism studies included one study with patients and no study with patients included in other traits.

**Table 8.** Voxel-wise effect sizes (hedge’s g) of the meta-regression results

Trait	Age	Sex	Scan	ImPros	Smooth	ThrCor	ICV	TGMV	Global	OtTrait	#CV	QA
N	0.0002 (0.00 – 0.00)	0.0421 (−0.43 – 0.51)	0.0052 (−0.07 – 0.07)	0.0092 (−0.15 – 0.15)	0.0005 (0.00 – 0.00)	0.0058 (−0.04 – 0.08)	0.0086 (−0.17 – 0.15)	0.0119 (−0.16 – 0.17)	0.0109 (−0.14 – 0.12)	0.0115 (−0.24 – 0.21)	0.0015 (−0.03 – 0.02)	0.0079 (−0.06 – 0.07)
E	0.0002 (0.00 – 0.00)	0.0403 (−0.19 – 0.46)	0.0039 (−0.03 – 0.09)	0.0088 (−0.12 – 0.08)	0.0005 (0.00 – 0.00)	0.0059 (−0.05 – 0.05)	0.0102 (−0.14 – 0.09)	0.0140 (−0.15 – 0.14)	0.0108 (−0.09 – 0.13)	0.0116 (−0.05 – 0.20)	0.0020 (−0.02 – 0.02)	0.0085 (−0.11 – 0.08)
A	0.0002 (0.00 – 0.00)	0.0472 (−0.48 – 0.44)	0.0059 (−0.13 – 0.14)	0.0119 (−0.20 – 0.19)	0.0005 (0.00 – 0.00)	0.0070 (−0.14 – 0.11)	0.0140 (−0.26 – 0.26)	0.0154 (−0.17 – 0.18)	0.0132 (−0.18 – 0.19)	0.0120 (−0.25 – 0.25)	0.0016 (−0.02 – 0.02)	0.0114 (−0.07 – 0.05)
C	0.0002 (0.00 – 0.00)	0.0354 (−0.36 – 0.33)	0.0040 (−0.11 – 0.12)	0.0073 (−0.18 – 0.18)	0.0004 (0.00 – 0.00)	0.0067 (−0.10 – 0.10)	0.0083 (−0.21 – 0.21)	0.0094 (−0.19 – 0.18)	0.0092 (−0.16 – 0.19)	0.0088 (−0.22 – 0.24)	0.0019 (−0.02 – 0.03)	0.0052 (−0.09 – 0.09)
O	0.0002 (0.00 – 0.00)	0.0435 (−0.27 – 0.61)	0.0039 (−0.03 – 0.04)	0.0085 (−0.08 – 0.16)	0.0039 (−0.03 – 0.04)	0.0076 (−0.05 – 0.09)	0.0087 (−0.09 – 0.10)	0.0150 (−0.06 – 0.19)	0.0093 (−0.08 – 0.19)	0.0088 (−0.08 – 0.15)	0.0150 (−0.06 – 0.19)	0.0105 (−0.21 – 0.04)

*Note.* The reporting range of the effect size is rounded to 2 decimals, and therefore, some values are displayed as 0.00; however, all voxels have non-zero effect sizes. A, Agreeableness; Age, mean age of the studied sample; C, Conscientiousness; E, Extraversion; Global, the inclusion of any global brain indices (ICV, TGMV, TBV) as covariate (study that did not include any global indices as covariate as the reference); ICV, the inclusion of ICV as covariate (study that did not include ICV as covariate as the reference); ImPros, image processing method (VBM (reference) *versus* SBM); N, Neuroticism; O, Openness; OtTrait, the inclusion of other Big Five personality traits as covariate (study that did not include other personality traits as covariate as the reference); Scan, scanner magnetic field strength; Sex, percentage of females in the study; Smooth, smooth kernel; QA, quality score; TGMV, the inclusion of TGMV as covariate (study that did not include TGMV as covariate as the reference); ThrCor, threshold correction (uncorrected *versus* cluster-level correction *versus* voxel-/vertex-level correction); #CV, total number of covariates.

## 3. Discussion

MRI studies have come under criticism for reporting under-powered and non-replicable findings (Button et al., 2013; Szucs & Ioannidis, 2017). Here, we used a systematic review and a meta-analysis approach to discern which findings, if any, would replicate reported associations between personality and brain measures. Surprisingly, we found no evidence for robust associations between any of the Big Five traits and brain structural indices (i.e., GMV, CT, and SA). Although we observed some consistent results from the qualitative systematic review, these findings failed confirmation when we used a quantitative meta-analytic approach.

### 3.1. Comparison with previous systematic review and/or meta-analysis studies

To our knowledge, only three studies used a systematic review and/or quantitative meta-analytic approach to evaluate the replicability of associations between personality traits and brain structural indices. One meta-analysis (Mincic, 2015) examined the association between GMV and a broad composite meta-trait named “negative emotionality” and included studies that measured one of these traits: Behavioral inhibition, harm avoidance, trait anxiety, or neuroticism.

Two other studies restricted their analyses to single “Big Five” traits only. One review by Montag, Reuter and colleague (2013) focused on GMV and neuroticism. These investigators reported heterogeneous findings across studies but noted consistent negative associations with neuroticism in prefrontal regions that included SFG and MFG and the OFC. This observation is consistent with our systematic review. However, Montag and colleagues did not subject their reviewed studies to a quantitative meta-analysis to determine the robustness of this observation, whereas our meta-analysis failed to confirm this observed association.

The second study was conducted by Lai et al. (2019) to examine the association between GMV and extraversion, using both a systematic review and meta-analytic approach. Based on quantitative meta-analysis, these investigators reported positive associations in the medOF and PRC, and negative associations in PHC, SMG, ANG, and MFG. The results contradicted our null meta-analysis result for extraversion. The discrepancies might derive from, first, the difference in the studies that were included across the two meta-analyses. First, Lai et al. (2019) only included VBM studies, whereas the present study included both VBM (*n* = 14) and SBM (*n* = 4) studies. Thus, four studies using SBM were not included in Lai et al. (2019). Second, Grodin and White (2015) were excluded from the present study due to the personality instrument this study used. This study used two subscales (Social Potency and Social Closeness) from the Multidimensional Personality Questionnaire Brief Form as proxy of extraversion. However, we determined that this instrument does not align with the same conceptual structure of the global extraversion of the Big Five and therefore excluded this study. Third, two studies that used different image processing were by DeYoung et al. (2010) (which did not perform segmentation in the preprocessing) and by Yasuno et al. (2017) (which used a T1w/T2w ratio signal), and were not included in Lai et al. (2019). In addition, Nostro et al. (2017), which was included in Lai et al. (2019), were excluded from the present study due to non-independent samples overlapping with another larger study (Owens et al., 2019). The second difference between the present study and Lai et al. (2019) is the meta-analytic software versions used. Lai et al. (2019) used the previous version of SDM, Anisotropy Effect-Size Seed-based *d* Mapping (AES-SDM) (Radua et al., 2012, 2014), whereas the present study used the latest version, SDM-PSI. The major improvement of SDM-PSI is the implementation of multiple imputations of study images to avoid the bias from the single imputation and a less biased estimation of population effect size, and it is considered more robust than AES-SDM (Albajes-Eizagirre, Solanes, Vieta, et al., 2019).

### 3.2. Possible explanations of heterogeneous findings

Several possible explanations may account for discrepancies across the studies, including, but not limited to, (1) sample heterogeneity, (2) Big Five personality instruments, (3) structural image data acquisition, processing, and analytic strategies, (4) statistical approach and statistical significance threshold, and (5) the heterogeneous nature of personality and brain structure. The following sections discuss the above-listed factors in greater detail.

#### 3.2.1. Sample heterogeneity

Sample characteristics that potentially contribute to highly heterogeneous results across the literature include mean age and age range, sex, and the inclusion of patient cohorts and different levels of personality traits across samples.

##### 3.2.1.1. Age

**Figure 4.**
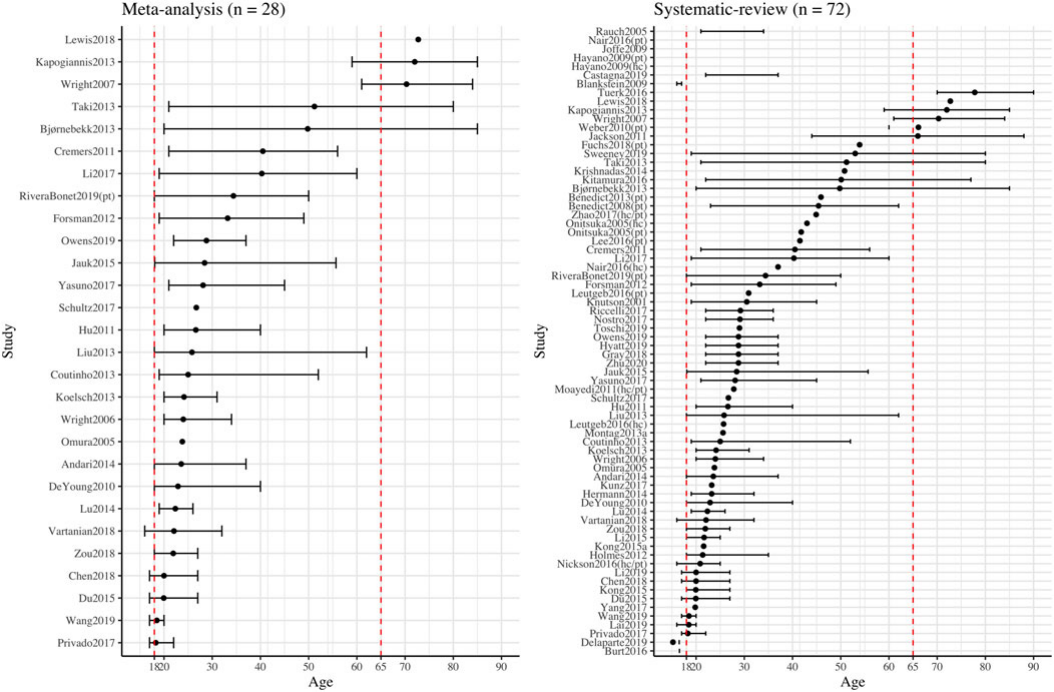
Sample Mean Age and Age Range Distribution of Studies Included in the Systematic Review and Meta-analysis across Big Five Personality Traits and Three Brain Indices. *Note.* The study (y-axis) was ordered by the mean age (dot) from each study. Studies were separately labeled as “(hc)/(pt)/(hc/pt)” indicating results from the given study were reported separately for healthy and patient groups or combining healthy and patient groups. Not all studies provided the information for mean age or age range, thus, data from those studies were presented incompletely or not presented. Two red dashed vertical lines indicating the age of 18 and 65

From the systematic review, age correlated negatively with neuroticism, extraversion, and openness, but positively with agreeableness (note that not all studies that examined association between age and personality traits reported significant associations, as shown in Table S18). Those qualitative observations are consistent with previous population-based cross-sectional and mean-level studies (Allemand et al., 2007; Donnellan & Lucas, 2008). We examined whether age could account for heterogeneous meta-analysis results for all five traits using meta-regression, but we did not observe any significant age effect on any of the meta-analyses for five traits. However, the lack of a significant effect may be due, in part, to a narrow age range across the samples, which mainly consisted of adults aged 18 – 40 (Figure 4). This may hinder the generalizability of the results. Only one study (Nickson et al., 2016) examined longitudinal changes of personality traits and two studies (Nickson et al., 2016; Taki et al., 2013) examined longitudinal changes of GMV. Nickson et al. (2016) observed no association between AMY GMV changes and neuroticism and extraversion changes over an average of two-year interval among a mixed sample of patients with major depressive disorder and healthy controls. However, considering the small-to-moderate sample size and heterogeneous composition of the sample, future research with longitudinal study design is required to explore the causal relationship between age, personality traits, and brain structures. Furthermore, none of the included studies considered non-linear associations between age and personality traits, despite evidence in support of such a relation (Donnellan & Lucas, 2008; Terracciano et al., 2005). For example, a curvilinear association was reported between age and conscientiousness, such that the highest scores were observed in middle adulthood (Donnellan & Lucas, 2008). Future research with the consideration of non-linear nature of age and personality traits is required to delineate the relationship.

##### 3.2.1.2. Sex

From our systematic review, females reported higher levels of personality measures across all five traits, with the exception of extraversion from Omura et al. (2005) and openness from Gray et al. (2018) (note that not all studies that examined sex difference in personality traits reported significant difference, as shown in Table S19), and this observation is consistent with a previous population-based cross-sectional study (Soto et al., 2011). Interestingly, we observed that the mean ages of the participants were younger in Omura et al. (2005) and Gray et al. (2018), compared to other studies that reported higher levels of extraversion and openness in females. This observation is also partially consistent with the observations from Soto et al. (2011), which showed that, on average, sex differences in personality traits vary across difference ages. Among all included studies in the systematic review (across five traits and three brain indices), 14 studies examined sex-dependent associations between the Big Five and brain structure. Two main approaches were used, including conducting trait–brain analysis separately for females and males and conducting sex-by-trait interaction analysis. As summarized in the previous section, the results are inconsistent across those studies, such that some studies reported trait–brain associations only in females (Hu et al., 2011), only in males (Hu et al., 2011; Montag, Eichner, et al., 2013; Nostro et al., 2017), or in neither group (Knutson et al., 2001; Liu et al., 2013; Wright et al., 2006, 2007), and interaction analyses produced similarly conflicting results ((Blankstein et al., 2009; Cremers et al., 2011; Sweeney et al., 2019) *versus* (Bjørnebekk et al., 2013; J. C. Gray et al., 2018; Lewis et al., 2018; Wang et al., 2019)). We then examined whether sex (using the proportion of females) could account for heterogeneous meta-analysis results for all five traits using meta-regression, but again we did not observe significant effects. The potential explanations for sex difference include sex-related hormonal variability (De Vries, 2004), early biological and social developmental trajectory (Blankstein et al., 2009; Goldstein et al., 2001), and social processing differences in response to the environment (Wager et al., 2003). However, due to the mixed results from the literature, future research should take sex difference into account when examining the association between personality traits and brain structures.

##### 3.2.1.3. Inclusion of patient cohorts and different levels of personality traits across samples

From the systematic review studies (across five traits and three brain indices), 14 studies included patient cohorts, as summarized in Table S17. Most studies reported higher mean level of neuroticism and lower mean level of extraversion and conscientiousness in patient cohorts, compared to healthy individuals (note that not all studies reported group differences in these three traits, as shown in Table S17), whereas no mean-level differences were reported for agreeableness and openness. Among those 14 patient cohort studies, three studies reported group differences in trait–brain associations, noting opposite associations between patients and healthy participants. It is possible that different levels of personality traits between patient and healthy groups contribute to the conflicting trait–brain associations. For example, a higher mean level of neuroticism was observed among patients with alcohol use disorder, compared to healthy participants (Zhao et al., 2017). However, no group mean-level difference was observed in Nair et al. (2016) and Moayedi et al. (2011). Alternative explanations include symptoms associated with the given medical or psychiatric conditions and brain structural differences underlying those conditions. To remove the potential effect from patient cohorts for meta-analysis, we conducted a sub-group meta-analysis excluding patient cohort studies and the result from neuroticism (this is the only trait that with patient cohort study in meta-analysis) remained unchanged.

Considering the potential influence of levels of personality traits across studies among non-patient studies, we compared the mean scores of personality trait measures among systematic review studies that reported contradictory associations. For example, two included studies reported associations in opposite directions between openness and PCC GMV, with higher mean level of openness reported in Yasuno et al. (2017) compared to Kitamura et al. (2016). On the other hand, another two studies that reported associations in opposite directions between extraversion and MFG GMV reported comparable mean-level extraversion scores (Blankstein et al., 2009; Coutinho et al., 2013). However, due to differences in personality trait instruments and scoring methods (e.g., some studies reported raw score, whereas some studies reported T scores), lacking reporting of personality scores in some studies, it is difficult to determine whether the levels of personality traits might play a role in conflicting results we observed from the included studies.

#### 3.2.2. Heterogeneity in big five trait instruments

The use of different personality trait instruments might contribute to discrepancies across included studies. The most commonly used instruments were the Neuroticism, Extraversion, Openness Personality Inventory – Revised (NEO-PI-R) and the NEO-Five-Factor Inventory (NEO-FFI, short version of NEO-PI-R). Other instruments included the International Personality Item Pool (IPIP), Eysenck Personality Questionnaire (EPQ), Big Five Inventory (BFI), Big Five Structure Inventory (BFSI), Big Five Aspects Scale (BFAS), and 16 Personality Factor test (16 PF). Although studies showed high correlations between different instruments, some trait scales showed only low-to-moderate correlations (Gow et al., 2005). We examined whether the use of different instruments (NEO *versus* non-NEO) could account for heterogeneous meta-analysis results using sub-group analysis with only studies using NEO (either NEO-PI-R or NEO-FFI), but we did not observe significant results for all five traits. In addition, all the instruments listed were self-report questionnaires. Studies suggested combined observation- and interview-based, informant report (Connolly et al., 2007; Hyatt et al., 2019) or to use physiological responses (Taib et al., 2020) to better capture the complex construct of personality and avoid the bias from self-report.

#### 3.2.3. Heterogeneity in structural image data acquisition, processing, and analytic strategies

The heterogeneities of the structural image data acquisition, (pre)processing, and analytic strategies may also have contributed to discrepancies across studies.

##### 3.2.3.1. Structural image data acquisition and processing

The use of different MRI scanners, scanner magnetic field strength, voxel size, and smoothing kernel could result in differences in image spatial resolution and signal-to-noise ratio. In addition, the use of VBM *versus* SBM processing methods might lead to inconsistent results. Although Kunz et al. (2017) reported highly correlated total GMV results between VBM and SBM processing methods, none of the included studies directly compared VBM and SBM for regional structural results. However, the small number (18 VBM and 5 SBM studies across five traits) of studies we could include in the meta-regression limits any strong conclusions.

##### 3.2.3.2. Structural image data analytic approaches

Different levels of structural analysis, whole-brain *versus* region-of-interest (ROI), could contribute to the heterogeneous results. Note that only studies using whole-brain voxel-/vertex-wise analysis *and* the same threshold across the whole brain were included in the meta-analysis to avoid the bias derived from regions with more liberal threshold (i.e., *a prior* ROI analysis) (Albajes-Eizagirre, Solanes, Fullana, et al., 2019; Q. Li et al., 2020), which makes a direct comparison between whole-brain and ROI studies difficult. Li et al. (2017) made a direct comparison between whole-brain vertex-wise and whole-brain regional parcellation-based analyses and reported inconsistent results (i.e., different traits were associated with different brain indices in different regions) from the same group of participants. The authors suggested that the two approaches provide different advantages, such that vertex-wise approach could potentially give more accurate localizations, whereas parcellation-based approach could potentially achieve higher test-retest reliability across populations/studies. Furthermore, the selection of the atlas to label brain region for a given peak coordinates (for whole-brain voxel-/vertex-wise studies) or to extract mean value for pre-defined ROIs (for ROI studies) adds another layer of heterogeneity, as the same voxel/vertex coordinates may be labeled differently across atlases. Atlas used by the included studies can be found in Supplementary Tables S2 - S16. Future research should utilize alternative approaches, such as voxel-/vertex-based and parcellation-based approaches, to evaluate the reliability of the results.

The present study was limited to studies examining brain structure using T1-weighted structural MRI. Alternatively, brain structure can be measured by diffusion MRI. By measuring diffusivity of the water molecules, diffusion imaging allows an indirect way to measure white matter fiber structure (Mori & Zhang, 2006) and it has been implemented in the field of personality neuroscience (e.g., Avinun et al., 2020; Bjørnebekk et al., 2013; Privado et al., 2017; Ueda et al., 2018; Xu & Potenza, 2012). Furthermore, beyond single voxel/vertex and single parcellated region, connectome and network approaches may offer promising alternative ways to investigate *patterns* of brain structures and their associations with personality (Markett et al., 2018). Network approaches not only measure characteristics of nodes (brain regions) and edges (connections between brain regions) within and between the brain networks but also measure the local and global organization of the brain networks (Sporns & Zwi, 2004). An optimal connectome approach may be achieved by implementing both high-resolution structural and diffusion MRI images (Gong et al., 2012; Sporns et al., 2005).

#### 3.2.4. Heterogeneity in statistical approach and statistical significance threshold

The use of different statistical approaches and statistical significance thresholds might contribute to discrepancies across studies.

##### 3.2.4.1. Covariates in model specification

For model specification, commonly used covariates include age, sex, and global brain indices. Among the included studies in the systematic review (across five traits and three brain indices), covariates included age (*n* = 55 studies), sex (*n* = 47), TGMV/mean CT (*n* = 13), total brain volume (TBV) (*n* = 9), intracranial volume (ICV) (*n* = 26). Other covariates included intelligence (*n* = 7) and education (*n* = 3). Studies have directly examined influence of the covariates and suggested that the associations between personality traits and brain structures change dramatically (Hu et al., 2011; Hyatt et al., 2020). For example, Hu et al. (2011) reported different trait–GMV associations when controlling for different combinations of age, sex, and TGMV, and Hyatt et al. (2020) reported remarked changes from the inclusion of ICV as covariate in statistical significance of the relation between various psychological variables (e.g., personality, psychopathology, cognitive processing) and regional GMV. In addition to demographic covariates, some studies also controlled for other personality traits (*n* = 15 across five traits among systematic review studies). Statistically speaking, the “unique association” of a given trait by including the other traits as covariates seemed to be reasonable, but whether the inclusion of other traits as covariates is still under debate, as some studies argued that the interpretation of “partial association” might not be straightforward (Lynam et al., 2006; Sleep et al., 2017) and might fail to capture the inter-correlations between personality traits (e.g., Gray et al., 2018; Holmes et al., 2012; Liu et al., 2013). Profile- or cluster-based approaches have been proposed as an alternative way to capture the inter-dependency of personality traits (e.g., Gerlach et al., 2018; Y. Li et al., 2020; Mulders et al., 2018). To assess whether the inclusion of different covariates could account for heterogeneous meta-analysis results, we conducted a series of meta-regression analyses with the inclusion of (1) ICV, (2) TGMV, (3) any global brain indices (ICV, TGMV, or TBV), (4) other personality traits as covariates, and (5) the total number of covariates. Our results did not change as a function of any of these variables, although this may also reflect the small number of studies that fulfilled relevant selection criteria. We suggest that future research and future synthesis work should take the inclusion of covariates into account.

##### 3.2.4.2. Significance threshold and multiple comparison correction

The choice of statistical significance threshold for reporting results should also be considered. Various levels of threshold were used among the included studies, including uncorrected *versus* corrected for multiple comparison, voxel-/vertex-level *versus* cluster-level correction, and different multiple comparison correction methods (e.g., family-wise error, Monte Carlo simulated, non-stationary, false discovery rate). Meta-analytic null results from this study may be due, in part, to positive results from studies that applied liberal statistical thresholds to data with small effect sizes, which were not robust enough to be replicable.

#### 3.2.5. Heterogeneous nature of personality and brain structure

##### 3.2.5.1. Replication challenges

Direct replication efforts in studies of personality and brain structure remain scarce to date. Replication was directly assessed in Owens et al. (2019) by comparing results with their earlier study (Riccelli et al., 2017), using data from the same dataset (i.e., HCP). Owens et al. (2019) demonstrated that not all results were replicated from the replication sample or the full sample. The sample characteristics, personality and image data acquisition, and processing were almost identical across those two studies, suggesting that other explanations than differences in sample characteristic and methodologies should be considered, according to Owens et al. (2019).

##### 3.2.5.2. Heterogeneous nature and individual difference

Finally, we consider the complex and heterogeneous nature of both personality and brain structures. First, a large number of brain regions were reported to be associated with one or more personality traits, and this observation might suggest that personality is constructed by many small effects from different brain regions (M. Li et al., 2019; Montag, Reuter, et al., 2013; Owens et al., 2019). Second, most conclusions from the literature were drawn from group mean levels, which ignored individual differences. Studies have demonstrated the influences of individual differences on cross-sectional and longitudinal changes in personality traits (Allemand et al., 2007; Lüdtke et al., 2009). Both genetic and environment factors have been suggested to contribute to the heterogeneities. For example, heritability of personality and regional brain structures has been suggested to contribute to heterogeneous associations between the two (Nostro et al., 2017; Valk et al., 2020). On the other hand, ample research has also demonstrated that both personality and brain structure are susceptible to change by the environment and experiences (e.g., Montag, Reuter, et al., 2013; Roberts & Mroczek, 2008). Third, considering the highly heterogeneous nature and individual differences shaped by genetic and environment in personality traits, it is also possible that the global dimension of the Big Five personality traits is too broad to have the universal representation in the brain. Based on NEO-PI-R (Costa & McCrae, 1992), each of the five personality traits is constructed by six facets. Studies have demonstrated that some trait facets contribute stronger, relative to the remaining facets for a given global trait, to the association between a certain global trait and brain structure (Bjørnebekk et al., 2013; M. Li et al., 2019). However, only a few studies included in the systematic review conducted additional facet analysis. Future research is recommended to examine the facets and variances of personality traits and brain structures and studies with regular follow-up are needed to evaluate the longitudinal changes.

##### 3.2.5.3. Considerations of statistical approach and power

The highly heterogeneous nature of personality and brain structure also raises the concern of statistical power of the previous literature to detect reliable associations between psychological phenotype and brain structure (Masouleh et al., 2019). An important aspect of statistical power relates to the image data analytic approach used. Although a voxel- or vertex-based approach could potentially provide more precise localization (T. Li et al., 2017), this also raises the concern of overestimating the statistical effect based on a peak voxel/vertex (Allen & DeYoung, 2017; DeYoung et al., 2010). On the other hand, with the advantage of improving signal-to-noise ratio, improving test-retest reliability, and reducing the number of variables, the use of whole-brain parcellation-based approach has been increased (e.g., Eickhoff et al., 2018; Hyatt et al., 2019; T. Li et al., 2017). Future research should carefully weigh the advantages and limitations of different image analytic approaches and possibly report on the congruency of their findings across multiple analysis methodologies.

### 3.3. Does a meaningful relation between the big five and brain structure really exist?

Having addressed several plausible factors contributing to heterogeneous findings and replication failure, we also consider the possibility that there is no meaningful relation between the Big Five personality traits and brain structure. Indeed, consistent null-to-very-small associations between the Big Five personality and brain structures have been reported by recent large-scale studies with over 1,100 participants (Avinun et al., 2020; Gray et al., 2018). For example, Avinun et al. (2020) investigated both global and facet levels of Big Five personality with structural indices from whole-brain parcellation (cortical CT, cortical SA, and subcortical GMV) and reported only conscientiousness (*R* ^*2*^ = .0044) (and its dutifulness facet (*R* ^*2*^ = .0062)) showed a small association with regional SA in superior temporal region. A recent study by Hyatt et al. (2021) of different levels of personality (from meta-traits, global Big Five traits, facets, and individual NEO-FFI items) and different levels of structural measures (from global brain measures to regional cortical and subcortical parcellations) reported that even the largest association (between Intellect facet of openness and global brain measures) yielded a mean effect size that was less than .05 (estimated by *R* ^*2*^). As we discussed above, alternative ways to assess brain structural features, such as connectome and network approaches, exist, and these may be better suited to map correspondences to complex traits than traditional approaches.

Our review of the literature was limited to studies of the Big Five personality model, which emerged from lexical analyses. The model serves the descriptive purpose but is not necessary explanatory (Deyoung & Gray, 2009; Montag & Davis, 2018). Thus, there is no *a priori* reason why these constructs should map neatly onto biological systems, although the Big Five traits are associated with biologically based constructs such as Gray’s Reinforcement Sensitivity Theory (RST) and Behavioral Inhibition and Approach System (BIS/BAS) (McNaughton & Corr, 2004), Panksepp’s Affective Neuroscience Theory (ANT) (Montag et al., 2021; Montag & Davis, 2018), and the dimensions of extraversion and neuroticism in Eysenck’s model (Eysenck, 1967). For example, Vecchione and colleagues (2021), applied a latent-variable analysis approach to a sample of 330 adults who completed both Carver and White’s BIS/BAS and the Big Five Inventory. These authors found that BIS correlated with emotional stability (inverse of neuroticism) and that BAS correlated with extraversion, after controlling for higher-order factors. Moreover, Montag and Panksepp (2017) demonstrated that seven ANT primary emotional systems are respectively associated with at least one global dimension of the Big Five, such as FEAR, ANGER, and SADNESS with neuroticism, PLAY with extraversion, CARE and ANGER with agreeableness, and SEEKING with openness. Considering that Big Five model closely maps onto biological motivational and emotional systems, future work should include side-by-side comparisons and integrate across conceptual models of personality structure (such as Big Five, RST, and ANT) to provide a comprehensive picture of personality. This could be an iterative process by which future personality models would continue to be refined by neural data and guide the next generation of imaging and other biological (e.g., genetic) studies.

### 3.4. Limitations

Some limitations should be noted when interpreting the present systematic review and meta-analysis results. First, one of the major challenges of meta-analysis is the trade-off between meta-analysis power and homogeneity of the included studies (Müller et al., 2018). Although no study, to our knowledge, has empirically evaluated the minimal number of studies required for meta-analysis using SDM, Eickhoff et al. (2016) suggested that between 17 to 20 studies are required to achieve adequate power using activation likelihood estimator (ALE) meta-analysis, although whether this result is transferrable to SDM meta-analysis remains to be determined. From the present meta-analysis for five personality traits and GMV studies, we maximized the number of studies by including heterogeneous studies such that studies with patient cohorts, measuring GMD, using T1w/T2w ratio signals, and so on, and we demonstrated that the results remained unchanged with more homogeneous sub-group meta-analysis by excluding the abovementioned studies. Second, the present meta-analysis results were derived from the reported peaks, rather than raw data, and this limits our evaluation of variability within each individual study. Lastly, the present review only included peer-reviewed articles. “Grey literature,” which refers to studies not captured by traditional database and/or commercial publishers, should be also considered to avoid bias when synthesizing the literature (Cooper et al., 2009). Previous review has found that the peer-reviewed published works had average greater effects and more significant results, compared to unpublished works like theses and dissertations (McLeod & Weisz, 2004; Webb & Sheeran, 2006). It is therefore unlikely that our general conclusions of lacking associations between the Big Five and structural brain measures would be altered by the inclusion of unpublished studies. Future researchers are encouraged to include studies from various sources and to carefully evaluate the quality of all works to provide reliable review.

### 3.5. Implication and conclusion

To our knowledge, this is the first study to systematically evaluate the entire published literature of the association between the Big Five personality traits and three brain structural indices, using a combination of qualitative and quantitative approaches. Qualitative results suggested highly heterogeneous findings, and the quantitative results found no replicable results across studies. Our discussion pointed out methodological limitations, the dearth of direct replications, as well as gaps in the extant literature, such as limited data on trait facets, on brain-personality associations across the life span, and on sex differences.

When it comes to the relation of Big Five personality and structural brain measures, the field of Personality Neuroscience may have come to a crossroads. In fact, the challenge of finding meaningful and replicable brain–behavior relations is not unique to Big Five personality traits. The same challenge has also emerged in other psychological constructs, including, but not limited to, intelligence and cognition (e.g., attention, executive function), psychosocial processes (e.g., political orientation, moral), and psychopathology (e.g., anxiety, internalizing, externalizing) (Boekel et al., 2015; Genon et al., 2017; Marek et al., 2020; Masouleh et al., 2019). On the one hand, the lack of any significant associations discourages further efforts down this path, as resources may be better spent on following other leads. On the other hand, we suggested several ways to strengthen future work investigating personality–brain structure associations. Consilience may be attained by parallel processing: Expanding upon next-generation structural imaging and analysis approaches, while developing new models of personality informed by cutting-edge data prescribing biological constraints. This may be best accomplished by coming to a consensus as a field on how we can best strengthen methodological rigor and replicability and creating an incentive structure that rewards large-scale consortia building in parallel to smaller-scale creative innovations in methods and constructs.

## Supplementary material

For supplementary material accompanying this paper visit https://doi.org/10.1017/pen.2021.5. click here to view supplementary material
