# Supplementary material for: “Nothing to see here”: No structural brain differences as a function of the Big Five personality traits from a systematic review and meta-analysis
Source: Personal Neurosci. 2022 Aug 9;5:e8. doi: 10.1017/pen.2021.5 (PMC9379932; doi:10.1017/pen.2021.5)
Supplement: Supplementary file 1 [file pensup.zip › S2513988621000055sup002.docx]

**Supplementary Materials**

**Supplementary Tables**

Table S1. Checklist of Quality Assessment for Studies Included in the Meta-analysis

Tables S2 – S6. Details of Studies Included in the Systematic Review and Meta-Analysis for Big Five Personality Traits and Gray Matter Volume (uploaded in Excel document)

Tables S7 – S11. Details of Studies Included in the Systematic Review and Meta-Analysis for Big Five Personality Traits and Cortical Thickness (uploaded in Excel document)

* *Note*. Studies that met the meta-analysis criteria were labeled with “MA,” although no meta-analysis was conducted for cortical thickness studies.

Tables S12 – S16. Details of Studies Included in the Systematic Review and Meta-Analysis for Big Five Personality Traits and Surface Area (uploaded in Excel document)

* *Note*. Studies that met the meta-analysis criteria were labeled with “MA,” although no meta-analysis was conducted for surface area studies.

Table S17. Details of Patient Studies in the Systematic Review and Meta-Analysis (uploaded in Excel document)

Table S18. Details of Studies Examining Age Differences across Big Five Personality Traits and Three Brain Indices (uploaded in Excel document)

Table S19. Details of Studies Examining Sex Differences across Big Five Personality Traits and Three Brain Indices (uploaded in Excel document)

Table S20. Abbreviations Used in the Main Content and in the Supplementary Materials

**Table S1**

*Checklist of Quality Assessment for Studies Included in the Meta-analysis*

| Domain | Criterion Description |
| --- | --- |
| Sample | Participant inclusion/exclusion criteria were clearly stated and were evaluated prospectively; for studies only included healthy individuals, psychiatric and medical illnesses were excluded |
| Sample | Was the study sample information well described? Important variables (demographic and global brain measures) were controlled either via stratification or statistics  1 = if both demographic (e.g., age, sex, education, intelligence, socioeconomic status, etc.) and global brain measures (e.g., total brain/gray matter volume, intracranial volume, etc.)  0.5 = if only control for either demographic or global brain measures  0 = if did not control for any |
| Sample | Sample size: for single group  1 = *N* ≥ 50; 0.5 = 30 ≤ *N* < 50; 0 = *N* < 30 |
| Method | Measurements were clearly described so that they could be reproduced 1 = correct description of the measurement and with proper citation or clearly describe items and scoring  0.5 = no citation or clear description |
| Method | The imaging technique used (including processing) was clearly described so that it could be reproduced |
| Method | Scanner magnet strength  1 = 3T (or above); 0.5 = 1.5T; 0 < 1.5T |
| Method | MRI slice-thickness :  1 = thickness ≤ 3 mm; 0.5 = thickness > 3 mm |
| Method | Statistical analysis  1 = corrected for multiple comparison; 0.5 = uncorrected |
| Result | Statistical parameters for significant and important nonsignificant association were provided 0.5 = if the peaks coordinates and effect sizes (e.g., *t*, Z, *r*, or *p* values) were not provided |
| Discussion | Conclusions were consistent with the results obtained, consistency and/or discrepancy compared with literature, and the limitations (e.g., study design, methodology, analyses, etc.) were discussed |

**Table S20**

*Abbreviations Used in the Main Content and in the Supplementary Materials*

| General terms | |
| --- | --- |
| Cor | corrected result |
| F/M | Female/Male |
| fc | facet-only study |
| FFM | Five-Factor Model |
| l/r | left/right hemisphere |
| MA | meta-analysis study |
| n.d. | no difference |
| NA | not applicable |
| Neg | negative |
| NS | not specified |
| NSig | no significant result |
| Pos | positive |
| PRISMA | Preferred Reporting Items for Systematic reviews and Meta-Analyses guidelines |
| pt | patient study |
| SDM-PSI | seed-based d mapping with permutation of subject images software |
| SES | socioeconomic status |
| Data project | |
| ABIP | Aoba Brain Imaging Project |
| ADEPT | Adolescent Development of Emotions and Personality Traits |
| BLSA | Baltimore Longitudinal Study of Aging |
| BNU | project from Beijing Normal University, China |
| BRID | Brain Resource International Database |
| CHD | Ongoing project in Chengdu, China |
| CPLS | Cognition and Plasticity through the Life Span |
| CR | Cognitive Reserve study |
| ECP | Epilepsy Connectome Project |
| HCP | Human Connectome Project |
| LBC1936 | Lothian Birth Cohort 1936 |
| NESDA | Netherlands Study of Depression and Anxiety |
| NKI-RS | Nathan Kline Institute – Rockland Sample |
| PSoBiD | Psychological, social, and biological determinants of ill health |
| RANN | Reference Ability Neural Network |
| SBFS | Scottish Bipolar Family Study |
| SMAS | Sydney Memory and Ageing Study |
| SWU | Ongoing project in Southwest University, China |
| UGA | A lager project from University of Graz, Austria |
| Study sample | |
| ADL | adolescents |
| AUD | alcohol use disorders |
| BPD | borderline personality disorder |
| CD | Crohn's Disease |
| CPD | chronic pain disorder |
| EOD | early-onset depression |
| HC | healthy control |
| HR-MDD | familial high-risk who developed MDD |
| HR-well | familial high-risk who remained healthy |
| MDD | major depressive disorder |
| MS | multiple sclerosis |
| PD | panic disorder |
| PTN | painful trigeminal neuropathy |
| SCZ | schizophrenia |
| TLE | temporal lobe epilepsy |
| TMD | temporomandibular disorder |
| Big Five instrument | |
| 16PF | 16 Personality Factor Test |
| 50-IPIP | 50-item International Personality Item Pool |
| BFAS | Big Five Aspects Scale |
| BFI | Big Five Inventory |
| BFSI | Big Five Structure Inventory |
| EPQ-R | Eysenck Personality Questionnaire-Revised |
| EPQ-RSC | Eysenck Personality Questionnaire-Revised Short Scale for Chinese |
| EPQ-RSS | Eysenck Personality Questionnaire-Revised Short Scale |
| EPQ | Eysenck Personality Questionnaire |
| EPS-A | Eysenck Personality Scale – Adult |
| NEO-FFI | NEO-Five Factor Model |
| NEO-PI-R | NEO-Personality Inventory-Revised |
| Image data related | |
| AAL | automated anatomical labeling |
| BIS | BioImage Suite |
| Bonf | Bonferroni |
| BVR | brain volume ratio (relative to the "rest of the brain volume") |
| CCS | Connectome Computation System |
| CT | cortical thickness |
| FDR | false discovery rate |
| fMRI | functional magnetic resonance imaging |
| fROI | functional region of interest |
| FS SEG | FreeSurfer segmentation |
| FS | FreeSurfer |
| FSL | FMRIB Software Library |
| FWE | Family-Wise Error |
| FWHM | Full-Width at the Half Maximum |
| GMC/GMD | gray matter concentration/density |
| GMV | gray matter volume |
| HCP-PP | HCP preprocessing pipeline |
| ICV | intracranial volume |
| MCS | Monte Carlo simulation |
| MNI | Montreal Neurological Institute |
| NSTA | non-stationary |
| ROI | region of interest |
| SA | surface area |
| SBM | surface-based morphometry |
| SPM | Statistical Parametric Mapping |
| TAL | Talairach |
| TBV | total brain volume |
| TBVR | total brain volume ratio |
| TGMV | total gray matter volume |
| TGMR | total gray matter ratio |
| TSA | total surface area |
| Uncor | uncorrected threshold/result |
| VBM | voxel-based morphometry |
| WBA | whole-brain analysis |
| WBPAR | whole-brain parcellation |
| Brain region labels | |
| ACC/G | anterior cingulate cortex/gyrus |
| AINS | anterior insula |
| AMY | amygdala |
| ANG | angular gyrus |
| antOFC | anterior orbitofrontal cortex |
| ATC | anterior temporal cortex |
| BG | basal ganglia |
| blAMY | basolateral amygdala |
| BRS | brainstem |
| CAD | caudate |
| CAL | calcarine gyrus |
| cauMFG | caudal middle frontal gyrus |
| CING | cingulate |
| CNS | cuneus |
| CRB | cerebellum |
| dACC | dorsal anterior cingulate cortex |
| DCC | dorsal cingulate cortex |
| dHIP | dorsal hippocampus |
| DLPFC | dorsolateral prefrontal cortex |
| DMPFC | dorsomedial prefrontal cortex |
| ENT | entorhinal cortex |
| FP | frontal pole |
| FPFC | fronto-palor prefrontal cortex |
| FSF | fusiform gyrus |
| HIP | hippocampus |
| HSG | Heschl's gyrus |
| IFG | inferior frontal gyrus |
| infOFC | inferior orbitofrontal cortex |
| INS | insula |
| IOG | inferior occipital gyrus |
| IPL/G | inferior parietal lobule/gyrus |
| IPS | intraparietal sulcus |
| ITG | inferior temporal gyrus |
| LNG | lingual gyrus |
| LOG | lateral occipital gyrus |
| LOTG | lateral occipito-temporal gyrus |
| MCC/G | middle cingulate cortex/gyrus |
| medFG | medial frontal gyrus |
| medOFC | medial orbitofrontal cortex |
| medSFG | medial superior frontal gyrus |
| MFG | middle frontal gyrus |
| midOFC | middle orbitofrontal cortex |
| MOG | middle occipital gyrus |
| MPFC | medial prefrontal cortex |
| MTG | middle temporal gyrus |
| MTP | middle temporal pole |
| NAc | nucleus accumbens |
| OFC/G | orbitofrontal cortex/gyrus |
| PCC/G | posterior cingulate cortex/gyrus |
| PCL | paracentral lobule |
| PCN | precuneus |
| PFC | prefrontal cortex |
| PHC | parahippocampal gyrus |
| PLD | pallidum |
| POS | parieto-occipital sulcus |
| PRC | precentral gyrus |
| PSC | postcentral gyrus |
| PSVA | para-striate visual area |
| PUT | putamen |
| RCG | rectal gyrus |
| RLO | rolandic operculum |
| rosACC | rostral anterior cingulate cortex |
| rosMFG | rostral middle frontal gyrus |
| S1 | primary somatosensory cortex |
| SALN | salience network |
| sfAMY | superficial amygdala |
| SFS | superior frontal sulcus |
| sgACC | subgenual anterior cingulate cortex |
| SMA | supplementary motor area |
| SMG | supramarginal gyrus |
| SOG | superior occipital gyrus |
| SPL/G | superior parietal lobule/gyrus |
| STG | superior temporal gyrus |
| STS | superior temporal sulcus |
| supOFC | superior orbitofrontal cortex |
| THA | thalamus |
| TNCV | total neocortical volume |
| TP | temporal pole |
| venACC | ventral anterior cingulate cortex |
| VLPFC | ventrolateral prefrontal cortex |
| VMPFC | ventromedial prefrontal cortex |

**Bibliography for the Included Studies across the Big Five and Brain Structure**

Andari, E., Schneider, F. C., Mottolese, R., Vindras, P., & Sirigu, A. (2014). Oxytocin’s fingerprint in personality traits and regional brain volume. *Cerebral Cortex*, *24*, 479–486. https://doi.org/10.1093/cercor/bhs328

Benedict, R. H. B., Hussein, S., Englert, J., Dwyer, M. G., Abdelrahman, N., Cox, J. L., Munschauer, F. E., Weinstock-Guttman, B., & Zivadinov, R. (2008). Cortical atrophy and personality in multiple sclerosis. *Neuropsychology*, *22*, 432–441. https://doi.org/10.1037/0894-4105.22.4.432

Benedict, R. H. B., Schwartz, C. E., Duberstein, P., Healy, B., Hoogs, M., Bergsland, N., Dwyer, M. G., Weinstock-Guttman, B., & Zivadinov, R. (2013). Influence of personality on the relationship between gray matter volume and neuropsychiatric symptoms in multiple sclerosis. *Psychosomatic Medicine*, *75*, 253–261. https://doi.org/10.1097/PSY.0b013e31828837cc

Bjørnebekk, A., Fjell, A. M., Walhovd, K. B., Grydeland, H., Torgersen, S., & Westlye, L. T. (2013). Neuronal correlates of the five factor model (FFM) of human personality: Multimodal imaging in a large healthy sample. *NeuroImage*, *65*, 194–208. https://doi.org/10.1016/j.neuroimage.2012.10.009

Blankstein, U., Chen, J. Y. W. W., Mincic, A. M., McGrath, P. A., & Davis, K. D. (2009). The complex minds of teenagers: Neuroanatomy of personality differs between sexes. *Neuropsychologia*, *47*, 599–603. https://doi.org/10.1016/j.neuropsychologia.2008.10.014

Burt, K. B., Whelan, R., Conrod, P. J., Banaschewski, T., Barker, G. J., Bokde, A. L. W., Bromberg, U., Buchel, C., Fauth-Buhler, M., Flor, H., Galinowski, A., Gallinat, J., Gowland, P., Heinz, A., Ittermann, B., Mann, K., Nees, F., Papadopoulos-Orfanos, D., Paus, T., … Garavan, H. (2016). Structural brain correlates of adolescent resilience. *Journal of Child Psychology and Psychiatry*, *57*, 1287–1296. https://doi.org/10.1111/jcpp.12552

Castagna, P. J. (2019). Structure related to function: Prefrontal surface area has an indirect effect on the relationship between amygdala volume and trait neuroticism. *Brain Structure & Function*, *224*, 3309–3320. https://doi.org/10.1007/s00429-019-01974-x

Chen, C., Mao, Y., Luo, J., He, L., & Jiang, Q. (2018). Regional gray matter volume mediates the relationship between conscientiousness and expressive suppression. *Frontiers in Human Neuroscience*, *12*, 301. https://doi.org/10.3389/fnhum.2018.00301

Coutinho, J. F., Sampaio, A., Ferreira, M., Soares, J. M., & Gonçalves, O. F. (2013). Brain correlates of pro-social personality traits: A voxel-based morphometry study. *Brain Imaging and Behavior*, *7*, 293–299. https://doi.org/10.1007/s11682-013-9227-2

Cremers, H., van Tol, M. J., Roelofs, K., Aleman, A., Zitman, F. G., van Buchem, M. A., Veltman, D. J., & van der Wee, N. J. A. (2011). Extraversion is linked to volume of the orbitofrontal cortex and amygdala. *PLoS ONE*, *6*. https://doi.org/10.1371/journal.pone.0028421

Delaparte, L., Bartlett, E., Grazioplene, R., Perlman, G., Gardus, J., DeLorenzo, C., Klein, D. N., & Kotov, R. (2019). Structural correlates of the orbitofrontal cortex and amygdala and personality in female adolescents. *Psychophysiology*, *56*, e13376. https://doi.org/10.1111/psyp.13376

DeYoung, C. G., Hirsh, J. B., Shane, M. S., Papademetris, X., Rajeevan, N., & Gray, J. R. (2010). Testing predictions from personality neuroscience: Brain structure and the big five. *Psychological Science*, *21*, 820–828. https://doi.org/10.1177/0956797610370159

Du, X., Luo, W., Shen, Y., Wei, D., Xie, P., Zhang, J., Zhang, Q., & Qiu, J. (2015). Brain structure associated with automatic thoughts predicted depression symptoms in healthy individuals. *Psychiatry Research*, *232*, 257–263. https://doi.org/10.1016/j.pscychresns.2015.03.002

Forsman, L. J., de Manzano, O., Karabanov, A., Madison, G., & Ullen, F. (2012). Differences in regional brain volume related to the extraversion-introversion dimension--A voxel based morphometry study. *Neuroscience Research*, *72*, 59–67. https://doi.org/10.1016/j.neures.2011.10.001

Fuchs, T. A., Dwyer, M. G., Kuceyeski, A., Choudhery, S., Carolus, K., Li, X., Mallory, M., Weinstock-Guttman, B., Jakimovski, D., Ramasamy, D., Zivadinov, R., & Benedict, R. H. B. (2018). White matter tract network disruption explains reduced conscientiousness in multiple sclerosis. *Human Brain Mapping*, *39*, 3682–3690. https://doi.org/10.1002/hbm.24203

Gray, J. C., Owens, M. M., Hyatt, C. S., & Miller, J. D. (2018). No evidence for morphometric associations of the amygdala and hippocampus with the five-factor model personality traits in relatively healthy young adults. *PloS One*, *13*, e0204011. https://doi.org/10.1371/journal.pone.0204011

Gustin, S. M., Peck, C. C., Macey, P. M., Murray, G. M., & Henderson, L. A. (2013). Unraveling the effects of plasticity and pain on personality. *The Journal of Pain*, *14*, 1642–1652. https://doi.org/10.1016/j.jpain.2013.08.005

Haas, B. W., Ishak, A., Anderson, I. W., & Filkowski, M. M. (2015). The tendency to trust is reflected in human brain structure. *NeuroImage*, *107*, 175–181. https://doi.org/10.1016/j.neuroimage.2014.11.060

Hayano, F., Nakamura, M., Asami, T., Uehara, K., Yoshida, T., Roppongi, T., Otsuka, T., Inoue, T., & Hirayasu, Y. (2009). Smaller amygdala is associated with anxiety in patients with panic disorder. *Psychiatry and Clinical Neurosciences*, *63*, 266–276. https://doi.org/10.1111/j.1440-1819.2009.01960.x

Hermann, A., Bieber, A., Keck, T., Vaitl, D., & Stark, R. (2014). Brain structural basis of cognitive reappraisal and expressive suppression. *Social Cognitive and Affective Neuroscience*, *9*, 1435–1442. https://doi.org/10.1093/scan/nst130

Holmes, A. J., Lee, P. H., Hollinshead, M. O., Bakst, L., Roffman, J. L., Smoller, J. W., & Buckner, R. L. (2012). Individual differences in amygdala-medial prefrontal anatomy link negative affect, impaired social functioning, and polygenic depression risk. *Journal of Neuroscience*, *32*, 18087–18100. https://doi.org/10.1523/JNEUROSCI.2531-12.2012

Hu, X., Erb, M., Ackermann, H., Martin, J. A., Grodd, W., & Reiterer, S. M. (2011). Voxel-based morphometry studies of personality: Issue of statistical model specification-effect of nuisance covariates. *NeuroImage*, *54*, 1994–2005. https://doi.org/10.1016/j.neuroimage.2010.10.024

Hyatt, C. S., Owens, M. M., Gray, J. C., Carter, N. T., MacKillop, J., Sweet, L. H., & Miller, J. D. (2019). Personality traits share overlapping neuroanatomical correlates with internalizing and externalizing psychopathology. *Journal of Abnormal Psychology*, *128*, 1–11. https://doi.org/10.1037/abn0000391

Jackson, J., Balota, D. A., & Head, D. (2011). Exploring the relationship between personality and regional brain volume in healthy aging. *Neurobiology of Aging*, *32*, 2162–2171. https://doi.org/10.1016/j.neurobiolaging.2009.12.009

Jauk, E., Neubauer, A. C., Dunst, B., Fink, A., & Benedek, M. (2015). Gray matter correlates of creative potential: A latent variable voxel-based morphometry study. *NeuroImage*, *111*, 312–320. https://doi.org/10.1016/j.neuroimage.2015.02.002

Joffe, R. T., Gatt, J. M., Kemp, A. H., Grieve, S., Dobson-Stone, C., Kuan, S. A., Schofield, P. R., Gordon, E., & Williams, L. M. (2009). Brain derived neurotrophic factor Val66Met polymorphism, the five factor model of personality and hippocampal volume: Implications for depressive illness. *Human Brain Mapping*, *30*, 1246–1256. https://doi.org/10.1002/hbm.20592

Kapogiannis, D., Sutin, A., Davatzikos, C., Costa, P., & Resnick, S. (2013). The five factors of personality and regional cortical variability in the Baltimore Longitudinal Study of Aging. *Human Brain Mapping*, *34*, 2829–2840. https://doi.org/10.1002/hbm.22108

Kitamura, S., Yasuno, F., Yamamoto, A., Kazui, H., Kudo, T., Matsuoka, K., Kiuchi, K., Kosaka, J., Nagatsuka, K., Iida, H., & Kishimoto, T. (2016). A structural model of age, grey matter volumes, education, and personality traits. *Psychogeriatrics*, *16*, 46–53. https://doi.org/10.1111/psyg.12118

Knutson, B., Momenan, R., Rawlings, R. R., Fong, G. W., & Hommer, D. (2001). Negative association of neuroticism with brain volume ratio in healthy humans. *Biological Psychiatry*, *50*, 685–690. https://doi.org/10.1016/S0006-3223(01)01220-3

Koelsch, S., Skouras, S., & Jentschke, S. (2013). Neural correlates of emotional personality: A structural and functional magnetic resonance imaging study. *PLoS ONE*, *8*. https://doi.org/10.1371/journal.pone.0077196

Kong, F., Hu, S., Xue, S., Song, Y., & Liu, J. (2015). Extraversion mediates the relationship between structural variations in the dorsolateral prefrontal cortex and social well-being. *NeuroImage*, *105*, 269–275. https://doi.org/10.1016/j.neuroimage.2014.10.062

Kong, X., Wei, D., Li, W., Cun, L., Xue, S., Zhang, Q., & Qiu, J. (2015). Neuroticism and extraversion mediate the association between loneliness and the dorsolateral prefrontal cortex. *Experimental Brain Research*, *233*, 157–164. https://doi.org/10.1007/s00221-014-4097-4

Krishnadas, R., Palaniyappan, L., Lang, J., McLean, J., & Cavanagh, J. (2014). Psychoticism and salience network morphology. *Personality and Individual Differences*, *57*, 37–42. https://doi.org/10.1016/j.paid.2013.09.016

Kunz, L., Reuter, M., Axmacher, N., & Montag, C. (2017). Conscientiousness is negatively associated with grey matter volume in young APOE epsilon 4-Carriers. *Journal of Alzheimer’s Disease*, *56*, 1135–1144. https://doi.org/10.3233/JAD-160854

Lai, H., Wang, S., Zhao, Y., Qiu, C., & Gong, Q. (2019). Neurostructural correlates of optimism: Gray matter density in the putamen predicts dispositional optimism in late adolescence. *Human Brain Mapping*, *41*, 1459–1471. https://doi.org/10.1002/hbm.24888

Lee, Y. J., Kim, S., Gwak, A. R., Kim, S. J., Kang, S.-G., Na, K.-S., Son, Y.-D., & Park, J. (2016). Decreased regional gray matter volume in suicide attempters compared to suicide non-attempters with major depressive disorders. *Comprehensive Psychiatry*, *67*, 59–65. https://doi.org/10.1016/j.comppsych.2016.02.013

Leutgeb, V., Ille, R., Wabnegger, A., Schienle, A., Schoggl, H., Weber, B., Papousek, I., Weiss, E. M., & Fink, A. (2016). Creativity and borderline personality disorder: Evidence from a voxel-based morphometry study. *Cognitive Neuropsychiatry*, *21*, 242–255. https://doi.org/10.1080/13546805.2016.1182904

Lewis, G. J., Dickie, D. A., Cox, S. R., Karama, S., Evans, A. C., Starr, J. M., Bastin, M. E., Wardlaw, J. M., & Deary, I. J. (2018). Widespread associations between trait conscientiousness and thickness of brain cortical regions. *NeuroImage*, *176*, 22–28. https://doi.org/10.1016/j.neuroimage.2018.04.033

Li, M., Wei, D., Yang, W., Zhang, J., & Qiu, J. (2019). Neuroanatomical correlates of extraversion: A test–retest study implicating gray matter volume in the caudate nucleus. *NeuroReport*, *30*, 953–959. https://doi.org/10.1097/WNR.0000000000001306

Li, T., Yan, X., Li, Y., Wang, J., Li, Q., Li, H., & Li, J. (2017). Neuronal correlates of individual differences in the big five personality traits: Evidences from cortical morphology and functional homogeneity. *Frontiers in Neuroscience*, *11*, 414. https://doi.org/10.3389/fnins.2017.00414

Li, W., Li, X., Huang, L., Kong, X., Yang, W., Wei, D., Li, J., Cheng, H., Zhang, Q., Qiu, J., & Liu, J. (2015). Brain structure links trait creativity to openness to experience. *Social Cognitive and Affective Neuroscience*, *10*, 191–198. https://doi.org/10.1093/scan/nsu041

Liu, W. Y., Weber, B., Reuter, M., Markett, S., Chu, W. C., & Montag, C. (2013). The Big Five of Personality and structural imaging revisited: A VBM - DARTEL study. *NeuroReport*, *24*, 375–380. https://doi.org/10.1097/WNR.0b013e328360dad7

Lu, F., Huo, Y., Li, M., Chen, H. H., Liu, F., Wang, Y., Long, Z., Duan, X., Zhang, J., Zeng, L., & Chen, H. H. (2014). Relationship between personality and gray matter volume in healthy young adults: A voxel-based morphometric study. *PloS One*, *9*, e88763. https://doi.org/10.1371/journal.pone.0088763

Moayedi, M., Weissman-Fogel, I., Crawley, A. P., Goldberg, M. B., Freeman, B. V, Tenenbaum, H. C., & Davis, K. D. (2011). Contribution of chronic pain and neuroticism to abnormal forebrain gray matter in patients with temporomandibular disorder. *NeuroImage*, *55*, 277–286. https://doi.org/10.1016/j.neuroimage.2010.12.013

Montag, C., Eichner, M., Markett, S., Quesada, C. M., Schoene-Bake, J.-C., Melchers, M., Plieger, T., Weber, B., & Reuter, M. (2013). An interaction of a NR3C1 polymorphism and antenatal solar activity impacts both hippocampus volume and neuroticism in adulthood. *Frontiers in Human Neuroscience*, *7*, 243. https://doi.org/10.3389/fnhum.2013.00243

Nair, V. A., Beniwal-Patel, P., Mbah, I., Young, B. M., Prabhakaran, V., & Saha, S. (2016). Structural imaging changes and behavioral correlates in patients with Crohn’s Disease in remission. *Frontiers in Human Neuroscience*, *10*, 460. https://doi.org/10.3389/fnhum.2016.00460

Nickson, T., Chan, S. W. Y., Papmeyer, M., Romaniuk, L., Macdonald, A., Stewart, T., Kielty, S., Lawrie, S. M., Hall, J., Sussmann, J. E., McIntosh, A. M., & Whalley, H. C. (2016). Prospective longitudinal voxel-based morphometry study of major depressive disorder in young individuals at high familial risk. *Psychological Medicine*, *46*, 2351–2361. https://doi.org/10.1017/S0033291716000519

Nostro, A. D., Müller, V. I., Reid, A. T., & Eickhoff, S. B. (2017). Correlations between personality and brain structure: A crucial role of gender. *Cerebral Cortex*, *27*, 3698–3712. https://doi.org/10.1093/cercor/bhw191

Omura, K., Constable, R. T., & Canli, T. (2005). Amygdala gray matter concentration is associated with extraversion and neuroticism. *Neuroreport*, *16*, 1905–1908. https://doi.org/10.1097/01.wnr.0000186596.64458.76

Onitsuka, T., Nestor, P. G., Gurrera, R. J., Shenton, M. E., Kasai, K., Frumin, M., Niznikiewicz, M. A., & McCarley, R. W. (2005). Association between reduced extraversion and right posterior fusiform gyrus gray matter reduction in chronic schizophrenia. *The American Journal of Psychiatry*, *162*, 599–601. https://doi.org/10.1176/appi.ajp.162.3.599

Owens, M. M., Hyatt, C. S., Gray, J. C., Carter, N. T., MacKillop, J., Miller, J. D., & Sweet, L. H. (2019). Cortical morphometry of the five-factor model of personality: Findings from the Human Connectome Project full sample. *Social Cognitive and Affective Neuroscience*, *14*, 381–395. https://doi.org/10.1093/scan/nsz017

Privado, J., Román, F. J., Saénz-Urturi, C., Burgaleta, M., & Colom, R. (2017). Gray and white matter correlates of the Big Five personality traits. *Neuroscience*, *349*, 174–184. https://doi.org/10.1016/j.neuroscience.2017.02.039

Rauch, S. L., Milad, M. R., Orr, S. P., Quinn, B. T., Fischl, B., & Pitman, R. K. (2005). Orbitofrontal thickness, retention of fear extinction, and extraversion. *Neuroreport*, *16*, 1909–1912. https://doi.org/10.1097/01.wnr.0000186599.66243.50

Riccelli, R., Toschi, N., Nigro, S., Terracciano, A., & Passamonti, L. (2017). Surface-based morphometry reveals the neuroanatomical basis of the five-factor model of personality. *Social Cognitive and Affective Neuroscience*, *12*, 671–684. https://doi.org/10.1093/scan/nsw175

Rivera-Bonet, C. N., Hermann, B., Cook, C. J., Hwang, G., Dabbs, K., Nair, V., Forseth, C., Mathis, J., Allen, L., Almane, D. N., Arkush, K., Birn, R., Conant, L. L., DeYoe, E. A., Felton, E., Humphries, C. J., Kraegel, P., Maganti, R., Nencka, A., … Meyerand, M. E. (2019). Neuroanatomical correlates of personality traits in temporal lobe epilepsy: Findings from the Epilepsy Connectome Project. *Epilepsy & Behavior*, *98*, 220–227. https://doi.org/10.1016/j.yebeh.2019.07.025

Schultz, C. C., Warziniak, H., Koch, K., Schachtzabel, C., Gullmar, D., Reichenbach, J. R., Schlosser, R. G., Sauer, H., & Wagner, G. (2017). High levels of neuroticism are associated with decreased cortical folding of the dorsolateral prefrontal cortex. *European Archives of Psychiatry and Clinical Neuroscience*, *267*, 579–584. https://doi.org/10.1007/s00406-017-0795-9

Schutter, D. J. L. G., Koolschijn, P. C. M. P., Peper, J. S., & Crone, E. A. (2012). The cerebellum link to neuroticism: A volumetric MRI association study in healthy volunteers. *PloS One*, *7*, e37252. https://doi.org/10.1371/journal.pone.0037252

Schutter, D. J. L. G., Meuwese, R., Bos, M. G. N., Crone, E. A., & Peper, J. S. (2017). Exploring the role of testosterone in the cerebellum link to neuroticism: From adolescence to early adulthood. *Psychoneuroendocrinology*, *78*, 203–212. https://doi.org/10.1016/j.psyneuen.2017.01.009

Sweeney, M., Tsapanou, A., & Stern, Y. (2019). Regional cortical thickness and neuroticism across the lifespan. *Psychiatry Research: Neuroimaging*, *286*, 39–44. https://doi.org/10.1016/j.pscychresns.2019.03.005

Taki, Y., Thyreau, B., Kinomura, S., Sato, K., Goto, R., Wu, K., Kawashima, R., & Fukuda, H. (2013). A longitudinal study of the relationship between personality traits and the annual rate of volume changes in regional gray matter in healthy adults. *Human Brain Mapping*, *34*, 3347–3353. https://doi.org/10.1002/hbm.22145

Toschi, N., & Passamonti, L. (2019). Intra-cortical myelin mediates personality differences. *Journal of Personality*, *87*, 889–902. https://doi.org/10.1111/jopy.12442

Tuerk, C., Zhang, H., Sachdev, P., Lord, S. R., Brodaty, H., Wen, W., & Delbaere, K. (2016). Regional gray matter volumes are related to concern about falling in older people: A voxel-based morphometrics study. *The Journals of Gerontology: Series A*, *71*, 138–144. https://doi.org/10.1093/gerona/glu242

Vartanian, O., Wertz, C. J., Flores, R. A., Beatty, E. L., Smith, I., Blackler, K., Lam, Q., & Jung, R. E. (2018). Structural correlates of Openness and Intellect: Implications for the contribution of personality to creativity. *Human Brain Mapping*, *39*, 2987–2996. https://doi.org/10.1002/hbm.24054

Wang, S., Zhao, Y., Li, J., Wang, X., Luo, K., & Gong, Q. (2019). Brain structure links trait conscientiousness to academic performance. *Scientific Reports*, *9*, 12168. https://doi.org/10.1038/s41598-019-48704-1

Weber, K., Giannakopoulos, P., Delaloye, C., de Bilbao, F., Moy, G., Moussa, A., Rubio, M. M., Ebbing, K., Meuli, R., Lazeyras, F., Meiler-Mititelu, C., Herrmann, F. R., Gold, G., & Canuto, A. (2010). Volumetric MRI changes, cognition and personality traits in old age depression. *Journal of Affective Disorders*, *124*, 275–282. https://doi.org/10.1016/j.jad.2009.11.016

Wei, D., Du, X., Li, W., Chen, Q., Li, H., Hao, X., Zhang, L., Hitchman, G., Zhang, Q., & Qiu, J. (2015). Regional gray matter volume and anxiety-related traits interact to predict somatic complaints in a non-clinical sample. *Social Cognitive and Affective Neuroscience*, *10*, 122–128. https://doi.org/10.1093/scan/nsu033

Wright, C. I., Feczko, E., Dickerson, B., & Williams, D. (2007). Neuroanatomical correlates of personality in the elderly. *NeuroImage*, *35*, 263–272. https://doi.org/10.1016/j.neuroimage.2006.11.039

Wright, C. I., Williams, D., Feczko, E., Barrett, L. F., Dickerson, B. C., Schwartz, C. E., & Wedig, M. M. (2006). Neuroanatomical correlates of extraversion and neuroticism. *Cerebral Cortex*, *16*, 1809–1819. https://doi.org/10.1093/cercor/bhj118

Yang, J., Hou, X., Wei, D., Wang, K., Li, Y., & Qiu, J. (2017). Only-child and non-only-child exhibit differences in creativity and agreeableness: Evidence from behavioral and anatomical structural studies. *Brain Imaging and Behavior*, *11*, 493–502. https://doi.org/10.1007/s11682-016-9530-9

Yang, J., Liu, H., Wei, D., Liu, W., Meng, J., Wang, K., Hao, L., & Qiu, J. (2016). Regional gray matter volume mediates the relationship between family socioeconomic status and depression-related trait in a young healthy sample. *Cognitive, Affective, & Behavioral Neuroscience*, *16*, 51–62. https://doi.org/10.3758/s13415-015-0371-6

Yang, J., Yin, P., Wei, D., Wang, K., Li, Y., & Qiu, J. (2017). Effects of parental emotional warmth on the relationship between regional gray matter volume and depression-related personality traits. *Social Neuroscience*, *12*, 337–348. https://doi.org/10.1080/17470919.2016.1174150

Yasuno, F., Kudo, T., Yamamoto, A., Matsuoka, K., Takahashi, M., Iida, H., Ihara, M., Nagatsuka, K., & Kishimoto, T. (2017). Significant correlation between openness personality in normal subjects and brain myelin mapping with T1/T2-weighted MR imaging. *Heliyon*, *3*, e00411. https://doi.org/10.1016/j.heliyon.2017.e00411

Zhao, Y., Zheng, Z.-L., & Castellanos, F. X. (2017). Analysis of alcohol use disorders from the Nathan Kline Institute-Rockland Sample: Correlation of brain cortical thickness with neuroticism. *Drug and Alcohol Dependence*, *170*, 66–73. https://doi.org/10.1016/j.drugalcdep.2016.10.040

Zhu, X., Wang, K., Cao, A., Zhang, Y., & Qiu, J. (2020). Personality traits and negative affect mediate the relationship between cortical thickness of superior frontal cortex and aggressive behavior. *Neuroscience Letters*, *718*, 134728. https://doi.org/10.1016/j.neulet.2019.134728

Zou, L., Su, L., Qi, R., Zheng, S., & Wang, L. (2018). Relationship between extraversion personality and gray matter volume and functional connectivity density in healthy young adults: An fMRI study. *Psychiatry Research: Neuroimaging*, *281*, 19–23. https://doi.org/10.1016/j.pscychresns.2018.08.018
